# Cystathionine-γ-lyase drives antioxidant defense in cysteine-restricted IDH1-mutant astrocytomas

**DOI:** 10.1093/noajnl/vdab057

**Published:** 2021-04-09

**Authors:** Andrés Cano-Galiano, Anais Oudin, Fred Fack, Maria-Francesca Allega, David Sumpton, Elena Martinez-Garcia, Gunnar Dittmar, Ann-Christin Hau, Alfonso De Falco, Christel Herold-Mende, Rolf Bjerkvig, Johannes Meiser, Saverio Tardito, Simone P Niclou

**Affiliations:** 1 NORLUX Neuro-Oncology Laboratory, Department of Oncology, Luxembourg Institute of Health, Luxembourg, Luxembourg; 2 Quantitative Biology Unit, Luxembourg Institute of Health, Luxembourg, Luxembourg; 3 Cancer Research UK Beatson Institute, Glasgow, UK; 4 Institute of Cancer Sciences, University of Glasgow, Glasgow, UK; 5 National Center of Genetics, Laboratoire national de santé, Dudelange, Luxembourg; 6 Department of Neurosurgery, University of Heidelberg, Heidelberg, Germany; 7 Department of Biomedicine, University of Bergen, Bergen, Norway; 8 Cancer Metabolism Group, Department of Oncology, Luxembourg Institute of Health, Luxembourg, Luxembourg

**Keywords:** antioxidant defense, cysteine, glioma, glutathione, IDH mutation, transsulfuration pathway

## Abstract

**Background:**

Mutations in isocitrate dehydrogenase 1 or 2 (*IDH1/2*) define glioma subtypes and are considered primary events in gliomagenesis, impacting tumor epigenetics and metabolism. IDH enzyme activity is crucial for the generation of reducing potential in normal cells, yet the impact of the mutation on the cellular antioxidant system in glioma is not understood. The aim of this study was to determine how glutathione (GSH), the main antioxidant in the brain, is maintained in IDH1-mutant gliomas, despite an altered NADPH/NADP balance.

**Methods:**

Proteomics, metabolomics, metabolic tracer studies, genetic silencing, and drug targeting approaches in vitro and in vivo were applied. Analyses were done in clinical specimen of different glioma subtypes, in glioma patient-derived cell lines carrying the endogenous IDH1 mutation and corresponding orthotopic xenografts in mice.

**Results:**

We find that cystathionine-γ-lyase (CSE), the enzyme responsible for cysteine production upstream of GSH biosynthesis, is specifically upregulated in IDH1-mutant astrocytomas. CSE inhibition sensitized these cells to cysteine depletion, an effect not observed in IDH1 wild-type gliomas. This correlated with an increase in reactive oxygen species and reduced GSH synthesis. Propargylglycine (PAG), a brain-penetrant drug specifically targeting CSE, led to delayed tumor growth in mice.

**Conclusions:**

*W*e show that IDH1-mutant astrocytic gliomas critically rely on NADPH-independent de novo GSH synthesis via CSE to maintain the antioxidant defense, which highlights a novel metabolic vulnerability that may be therapeutically exploited.

**Key Points**

- CSE, a key enzyme for de novo Glutathione production, is specifically upregulated in IDH-mutant astrocytomas.
- CSE is required for the survival of IDH-mutant astrocytoma cells under cysteine depletion and regulates Glutathione synthesis in vitro and in vivo.
- Chemical inhibition of CSE affects the growth of IDH-mutant astrocytomas in the mouse brain.

**Importance of the Study**

Wild-type IDH enzymes are critically important for the generation of reducing potential in the cell, allowing the regeneration of glutathione, the main antioxidant in the brain. This raises the question how glioma cells with mutant IDH cope with the challenge to maintain glutathione levels. Here we show that cystathionine-γ-lyase (CSE), the enzyme responsible for de novo glutathione synthesis, is specifically upregulated in IDH-mutant astrocytomas and that these tumors are highly sensitive to inhibition of CSE under cysteine limitation. These data identify a specific metabolic vulnerability in IDH-mutant astrocytomas that is druggable, thereby opening novel therapeutic opportunities for this aggressive tumor type.

The identification of mutations in isocitrate dehydrogenase 1 or 2 (IDH1, IDH2) has dramatically improved our understanding of glioma genesis^1^ and led to a better delineation of glioma subtypes. Whereas most primary glioblastomas (GBM) harbor the wild-type enzyme (IDHwt), mutated IDH (IDHm) largely defines lower grade gliomas and secondary GBM. LGGs are further subdivided based on 1p19q codeletion for oligodendrogliomas (OD) and frequent mutations in *TP53* and *ATRX* for astrocytomas (AS).^2^ Gliomas most often display the R132H mutation in IDH1. The wildtype enzyme produces α-ketoglutarate (αKG) from isocitrate thereby generating NADPH and CO_2_, while the mutant converts αKG into D-2-hydroxyglutarate (D2HG) and oxidizes NADPH.^3^ As a main contributor of cytosolic NADPH, IDH1wt is crucial for redox homeostasis via recycling of glutathione (GSH), the main antioxidant in the brain.^4^ Up to 65% of total NADPH may be generated from this reaction in IDH1wt GBM, whereas this contribution is decreased in IDH1m gliomas.^5^ Moreover, several studies suggest that IDH1m enhances chemo-radiosensitivity through GSH depletion and ROS generation,^6–9^ yet it is currently not understood how GSH levels are maintained in these tumors.

We previously identified metabolic aberrations in phospholipid, energy and oxidative stress regulation in IDHm gliomas.^10^ Notably, despite a drop in the NADPH/NADP+ ratio, GSH levels were barely affected in IDH1m tumors, while enzymes related to cysteine metabolism and de novo GSH production such as cystathionine-β-synthase (CBS) and Glutamate-cysteine ligase catalytic subunit (GCLC) showed increased gene expression, suggesting that de novo GSH synthesis might be active in these tumors.^10^

Based on clinical glioma specimen and unique patient-derived cell lines carrying the endogenous IDH1 mutation, we report that IDH1m astrocytomas specifically rely on cystathionine-γ-lyase (CSE, also known as cystathionase, *CTH* gene) to increase their cysteine pool for de novo GSH synthesis. Furthermore, we show that the CSE inhibitor propargylglycine (PAG) leads to increased cytotoxicity at low cysteine levels in vitro, and affects tumor growth in vivo. Our data warrant further investigation on the therapeutic potential of CSE in IDH1m astrocytoma patients.

## Materials and Methods

### Clinical Samples

Clinical glioma samples were obtained from 22 patients (10 IDHwt GBM; 8 IDH1m AS; 4 IDH1m OD) from the Haukeland Hospital, Bergen, Norway. Tissue fragments were stereotactically sampled (25 mg) during the operation and snap frozen. Patient characteristics and molecular diagnosis are given in Supplementary Table 1. Sample collection was approved by the local ethics committee Haukeland University Hospital, Bergen (REK 2010/130–2) in line with the Declaration of Helsinki.

### Patient-Derived Glioma Cell Lines

Patient-derived cell lines were grown as 3D spheres in defined supplemented DMEM-F12 medium (Supplementary Methods and Supplementary Figure S1a).^11–14^ NCH644, NCH601, and NCH421k correspond to IDH1wt GBM, NCH1681, and NCH551b to IDH1m AS, NCH612 to IDH1m OD, based on molecular classification.^2^ Cysteine was titrated by serial BIT dilutions (Supplementary Table 2). To confirm that the toxic effect of PAG in low BIT/cysteine medium was due to the lack of cysteine, the cells were rescued with extracellular cysteine (Supplementary Figure S4c).^15^

### Proteomics Analysis

Protein extracts from all 6 glioma cell lines were digested and peptides were analyzed on a Q-Exactive HF mass spectrometer (Thermo Scientific) coupled with a Dionex Ultimate 3000 RSLC chromatography system operated in column switching modeMS. Details of analysis are given in Supplementary Methods.

### In Vitro Assays

Western blot, qPCR, gene knockdown, sphere size measurement, cytotoxicity assay, and ROS measurements are described in Supplementary Methods.

### Metabolite and Flux Analysis in Cell Lines

For metabolite analysis, cells were grown in 30 µM cysteine. 400 µM of L-Serine (U-^13^C_3_, 99%) (CLM-1574-H-0.1 Eurisotop) were added to the culture medium obtaining a 1:1 ratio of ^13^C_3_-Serine/natural Serine. Metabolites were isolated after 4 days. LC-MS analysis was performed as described.^16,17^ Metabolites were identified using an in-house library of exact mass and known retention time generated using commercial standards on the same LC-MS system. A pilot GC-MS-based approach to determine steady state levels of ^13^C_3_-serine in different mice tissues (Supplementary Figure S4b) was performed as described in ref. ^18^.

### In Vivo Experiments

NCH1681 cells were stereotactically injected into the frontal cortex of NSG mice (NOD.Cg-Prkdc^scid^ Il2rg^tm1Wjl^/SzJ) (*n* = 20) and tumor growth was monitored by MRI (FSE-T2 sequence, 3T MRI system, MR Solutions) as described previously.^19^ Two months after implantation, mice with tumors (size range 2–10 mm^3^) were selected and randomized into 2 groups (8 mice per group). PAG (35 mg/kg) or saline was given ip (5 times/week during 3 months). For in vivo flux analysis, 130 mg/kg/h U-^13^C_3_ serine was infused in the tail vein (2 mice/group, 4 h), plus 2 healthy mice without tumors. Brain, liver, kidney, and heart were dissected and for LC-MS analysis. All procedures were approved by the animal welfare structure of LIH and the national authorities responsible for animal experiments in Luxembourg under the reference LUPA 2020/15.

## Results

### IDH1m Astrocytomas Upregulate Cystathionine-γ-lyase (CSE)

**Figure 1.**
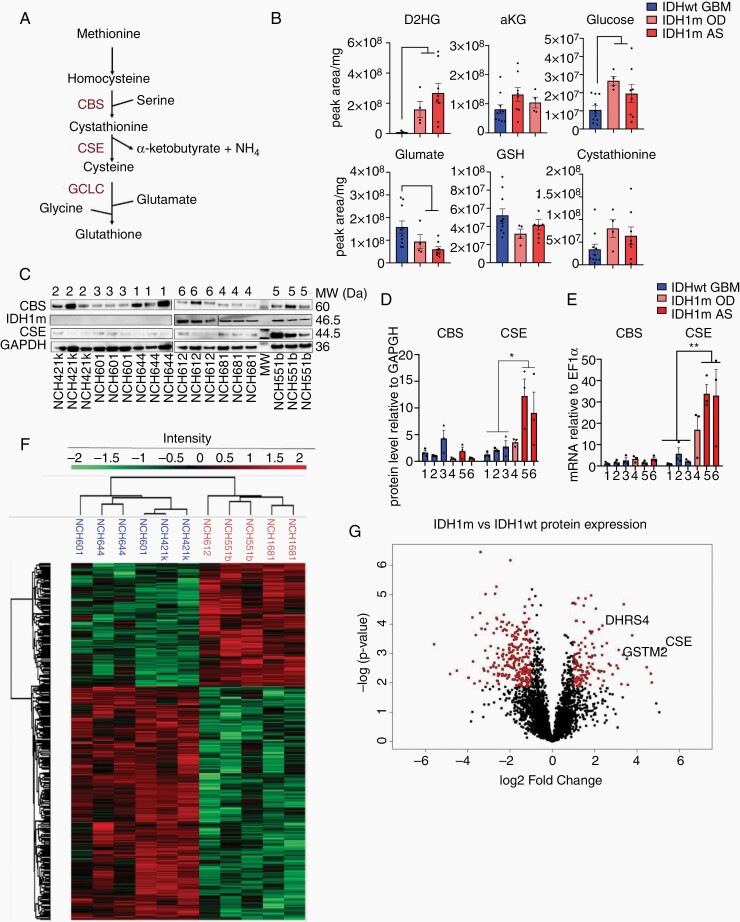
CSE is specifically upregulated in IDH1m astrocytomas. (A) Schematic of GSH *de novo* synthesis through the transsulfuration pathway. (B) Total levels of metabolites in 22 clinical samples, presented as peak area relative to mg of tissue. IDHwt (*n* = 10), IDH1m OD (*n* = 4), and IDH1m AS patients (*n* = 8). (C) Western blot for CBS, IDH1m, CSE, and GAPDH in IDH1wt and IDH1m cell lines. MW (Da) indicates specific molecular weight of each protein. (D) Quantification relative to GAPDH (*n* = 3). (E) *CBS* and *CSE* gene expression relative to *EF1α* determined by qPCR (*n* = 3). (F) Untargeted proteomic analysis of 3 IDH1wt and 3 IDH1m cell lines. Heat map representation of 575 differentially expressed proteins (false discovery rate [FDR] < 0.05). 127 proteins upregulated and 258 downregulated in IDH1m glioma cells (*n* = 2/cell line, except for NCH612 where *n* = 1). (G) Volcano plot of all 6265 detected proteins (in red differential proteins with FDR < 0.05, FC > 2). CSE, GSTM2, and DHRS4 are highlighted. For complete protein list, see Supplementary Table 3. D2HG, D2-hydroxyglutarate; αKG, α-ketoglutarate; GSH, glutathione; CSE, cystathionine-γ-lyase; CBS, cystathionine-β-synthase; GAPDH, Glyceraldehyde 3-phosphate dehydrogenase; IDH1wt, isocitrate dehydrogenase wild type; IDH1m, IDH1 mutant; AS, Astrocytoma; OD, Oligodendroglioma. Each independent dot of the bar charts corresponds to one biological replicate (*n*). Data presented as means ± SEM. *P < .05; ** *P* < .01; *** *P* < .001.

We previously reported the upregulation of CBS and GCLC in IDHm gliomas, suggesting an activation of the transsulfuration pathway (Figure 1A) in these tumors,^10^ an important source of cysteine for GSH synthesis in astrocytes.^20,21^ To corroborate this at the metabolite level, we analyzed specimen from 22 glioma patients including IDHwt GBM (*n* = 10), IDH1m OD (*n* = 4), and IDH1m AS (*n* = 8) (Supplementary Table 1). As expected, IDHm gliomas showed high D2HG and reduced αKG levels compared to IDHwt GBM (Figure 1B). Interestingly, while GSH levels were similar, we observed a decrease in glutamate and an increase in glucose and cystathionine in IDHm gliomas, supporting activation of the transsulfuration pathway (Figure 1B).

To address this in a model amenable to experimental manipulation, we turned to patient-derived glioma stem-like cells (GSCs) carrying the endogenous IDH1 mutation (NCH1681, NCH551b, NCH612) and control cells of IDHwt GBM (NCH644, NCH601, NCH421k) (Supplementary Figure S1a). IDH1m GSCs exhibited D2HG levels comparable to patients and display low proliferation rates.^11–13^ We confirmed the presence of the mutation at the genetic (Supplementary Figure S1b) and protein level (Figure 1C) and a more than 20-fold increase in D2HG (Supplementary Figure S1c). IDH1m allele frequency reached almost 100% in astrocytic cell lines (Supplementary Figure S1b), suggesting a loss of heterozygosity over time. The tendency to develop an IDH1m homozygosity in vitro has already been reported by others.^22^ Next, we investigated the expression of the main mediators of GSH production, CBS and cystathionine-γ-lyase (CSE) at the protein level. CSE is central to this pathway and the only known enzyme capable to synthesize cysteine, the limiting metabolite for GSH production.^23^ CSE generates cysteine, α-ketobutyrate and ammonia through the breakdown of cystathionine which is provided by CBS in the transsulfuration pathway (Figure 1A). While CBS showed similar expression in all tumor types, we found that CSE was upregulated in IDH1m cells both at the protein and mRNA level (Figure 1C–E). Among glioma subtypes, the upregulation of CSE was mainly seen in the IDH1m AS cell lines (NCH1681, NCH551b) (Figure 1C–E). This was confirmed in vivo in a panel of patient-derived orthotopic xenografts, while there was variable expression in IDHwt xenografts (Supplementary Figure S1d–f). We further validated these data interrogating our clinical material, which showed increased CSE protein in IDH1m tumors compared to IDHwt, with highest expression in AS (Supplementary Figure S1g,h). Gene expression data from public databases using the GlioVis portal, further confirmed these observations^15^ (Supplementary Figure S1i).

To obtain a more comprehensive view of protein deregulation in IDH1m gliomas, we performed label-free quantitative (LFQ) MS-based proteomics and identified a total of 6.265 proteins (Figure 1F). Principal component analysis (PCA) clearly separated 2 distinct populations based on IDH1 status (Supplementary Figure S1j). From the identified proteins, 575 were differentially expressed between IDH1wt and IDH1m cell lines (FDR < 0.05) (Figure 1F and Supplementary Table 3), with 127 proteins upregulated and 258 downregulated in IDH1m cells (FDR < 0.05, fold change [FC] >2; Figure 1G). Further analysis between AS and OD was not possible because only 1 OD sample was available. Of note, CSE was the most upregulated protein in IDH1m cells (FC = 37). In concordance with the above, this increase in CSE was mainly seen in AS cells (FC = 71). By gene ontology interrogation, we found that the top 10 processes of IDH1m upregulated proteins were linked to metabolism (Supplementary Figure S1k), while ribosome and RNA-related processes were downregulated in IDH1m cells (Supplementary Figure S1k), in line with the low proliferative phenotype of these tumors. Among the metabolic processes upregulated is the oxidation-reduction process including several proteins involved in redox regulation: glutathione S-transferase Mu 2 (GSTM2) in charge of GSH conjugation and dehydrogenase/reductase SDR family member 4 (DHRS4) an important NADPH producer (Figure 1G). In summary, we found that cystathionine accumulates in IDHm gliomas and identify its converting enzyme CSE and related partners to be specifically upregulated in IDH1m AS, suggesting an increased activity of the transsulfuration pathway in this glioma type.

### Loss of CSE Reduces Viability in IDH1m Astrocytoma Cells Under Cysteine Depletion

**Figure 2.**
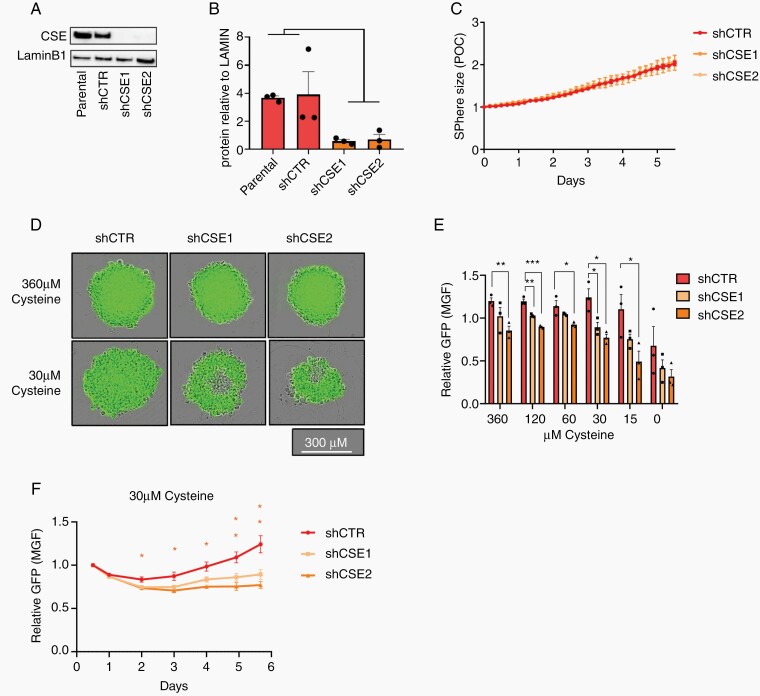
CSE is required to maintain viability of IDH1m astrocytoma cells under cysteine depletion. (A) CSE western blot analysis and (B) quantification relative to LaminB1 in IDH1m AS cells (NCH1681) with 2 CSE KD clones (shCSE1, shCSE2) compared to the parental and scramble control (shCTR) (*n* = 3). (C) Relative sphere size under standard culture conditions (360 µM cysteine). Data presented as fold change of phase object confluence (POC) of each cell line relative to day 0 (*n* = 3 with 5 spheres per experiment). (D) Representative fluorescent images of spheres after 6 days. (E) Relative GFP signal in CSE KD cells at decreasing cysteine concentrations (day 6). Measurements expressed as fold change of mean green fluorescence (MGF) of each cell line relative to starting point (360 µM cysteine at day 0.5 once the sphere was formed) (*n* = 3 with 5 spheres per experiment). (F) Time course of GFP signal of spheres at 30 µM cysteine. All data presented as means ± SEM. * *P* < .05, ** *P* < .01; *** *P* < .001.

To investigate the role of CSE in IDH1m AS, we established stable CSE knockdown (KD) lines in NCH1681 cells (IDH1m AS) using 2 different shRNAs. Both KD clones (shCSE1 and shCSE2) showed a strong drop in CSE protein (Figure 2A and B), yet we did not observe a significant difference in sphere size over time (Figure 2C) compared to control (shCTR), indicating that the proliferation capacity of these cells was unaltered under standard culture conditions. We argued that CSE may not be required if sufficient cysteine is provided from extracellular sources. In standard culture medium, the concentration of cysteine (360 µM combined cysteine and its oxidized dimer cystine; see Supplementary Methods) is higher than in human blood (80–180 µM)^24^ (source: HMDB). In contrast, in cerebrospinal fluid (CSF), a reference for nutrient values in the brain, cysteine is one of the lowest amino acids with values below 1 µM^25^ (source: HMDB). To reach a concentration more relevant to neural tissue, cysteine was serially diluted in cysteine-free DMEM. This led to a gradual loss of sphere viability (based on GFP fluorescence) in CSE KD cells (Figure 2D and E). Control cells only were affected upon complete cysteine withdrawal. The selective impact on CSE KD cells was most prominent at 30 µM cysteine, where the decrease in viability was already observed after 2 days of culture further increasing over time (Figure 2F). Taken together, these data provide evidence that under physiological cysteine concentrations, CSE is required to maintain viability of IDH1m AS cells.

### CSE Directs GSH Biosynthesis Upon Cysteine Starvation

**Figure 3.**
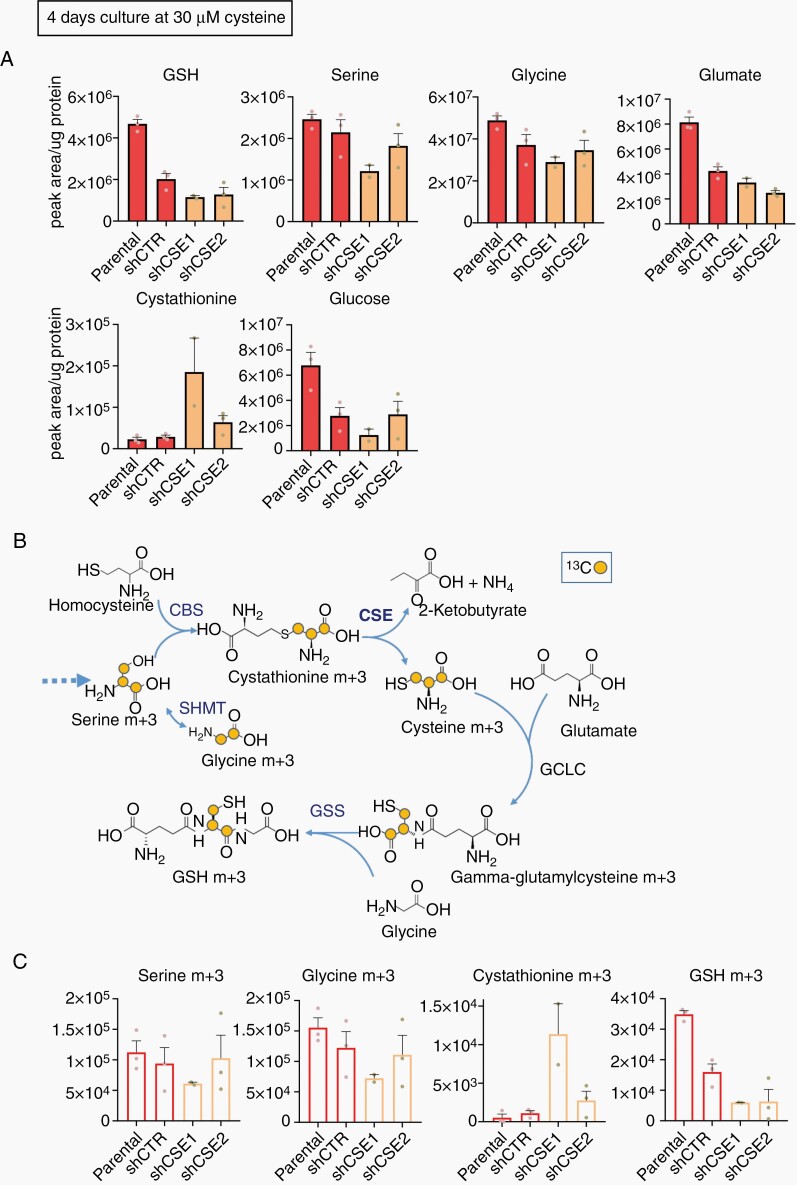
CSE directs GSH biosynthesis upon cysteine starvation. (A) Total metabolite level represented as normalized peak areas at 4 days in 30 µM cysteine. (B) Schematic of ^13^C_3_-serine metabolite flux toward GSH synthesis. (C) Isotopologues (normalized peak area) after ^13^C_3_-serine tracing at 4 days in 30 µM cysteine (*n* = 1 with 3 technical replicates in (A) and (C), no statistical analysis). CBS, cystathionine β-synthase; SHMT, Serine hydroxymethyltransferase; CSE, cystathionine-γ-lyase; GCLC, Glutamate-cysteine ligase catalytic subunit; GSS, glutathione synthetase.

We next asked whether CSE was required to maintain the cysteine pool for GSH production and whether the observed loss of viability was caused by a decrease in GSH levels. We determined the contribution of CSE to the overall GSH pool, by quantifying metabolites in cultures with 30 µM cysteine at different time points. After a 3-day incubation, no effects of metabolites were observed (Supplementary Figure S2a), in line with the cell viability results (Figure 2F). However, at 4 days, we found a decrease of total GSH level in the CSE KD cells (Figure 3A). Presumably all remaining cysteine had been used up which was also reflected in cell viability. This was confirmed in a second experiment (Supplementary Figure S2b). Unfortunately because of the gradual exhaustion of cysteine, many cells were vulnerable at day 4 and not all datapoints could be recovered in these experiments. Interestingly, there was a tendency for decreased serine, glycine, glutamate, and glucose in CSE KD cells, along with a slight accumulation in cystathionine (Figure 3A, Supplementary Figure S2b), suggesting an attempt of the cells to compensate for the loss of GSH. Taken together, these data support the premise that GSH biosynthesis depends at least in part on CSE activity upon cysteine depletion.

To confirm that the drop in GSH in CSE knockdown cells was due to a decrease of de novo GSH synthesis, we traced 13C_3_-serine in cells cultured with 30 µM cysteine for 4 days (ratio^13^C_3_-serine/natural serine = 1:1) (Figure 3B). As aforementioned, the limiting culture conditions prevented to recover sufficient biological replicates to perform statistical analysis (Figure 3C and Supplementary Figure S2c, respectively). Comparable levels of ^13^C_3_-serine were observed in all cells (Figure 3C, Supplementary Figure S2c), whereas the natural serine isotopologue was reduced in the KDs (Supplementary Figure S2d). This drop in natural serine together with the decrease in total glucose (Figure 3A) suggests a preference for glucose-derived serine when there is a high demand for this metabolite. In fact, the enrichment of heavy carbon labeled serine did not exceed 10% of the total pool (Supplementary Figure S2e). Despite the low serine tracer incorporation, there was a tendency for lower ^13^C_2_-glycine in CSE KD cells (Figure 3C, Supplementary Figure S2c) suggesting reduced serine-to-glycine conversion. In addition, shCSE cells tended toward increased levels of ^13^C_3_-cystathionine and a consistent decrease in ^13^C_3_-GSH (Figure 3C, Supplementary Figure S2c). The ratios GSH m+3/Serine m+3 (Supplementary Figure S2f) was significantly lower in the CSE KDs. These data support the notion that under cysteine deprivation CSE is essential to generate cysteine, preferentially via the transsulfuration pathway using glucose-derived serine to sustain GSH biosynthesis.

### IDH1m Astrocytoma Cells Are Selectively Sensitive to CSE Inhibition

**Figure 4.**
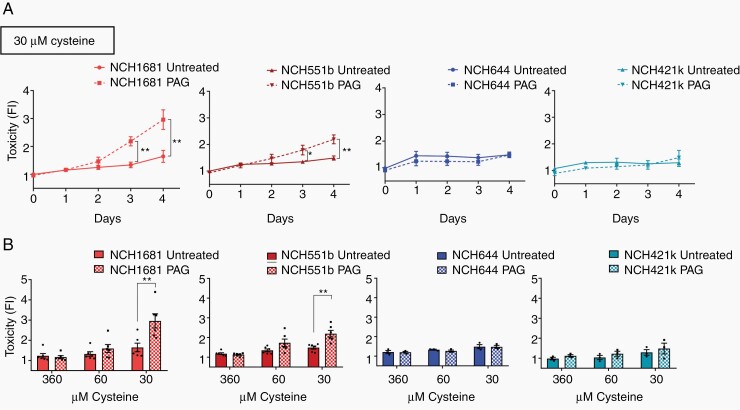
CSE inhibition selectively targets IDH1m astrocytoma cells under cysteine depletion. (A) Toxicity over time in IDH1m AS (NCH1681, NCH551b, in red, *n* = 6). and IDH1wt GBM (NCH644, NCH421k, in blue, *n* = 3) cells following PAG (10 mM) at 30 µM cysteine. Data presented as fold change of celltox (Promega®) probe fluorescence intensity (FI) relative to normal medium untreated (360 µM cysteine) at day 0. (B) Toxicity of PAG (10 mM) upon decreasing cysteine. Data presented as fold change relative to normal medium (360 µM cysteine) at day 0. IDH1m AS (*n* = 6). IDH1wt GBM (*n* = 3). Data presented as means ± SEM. * *P* < .05; ** *P* < .01.

Next, we wished to evaluate the therapeutic potential of CSE inhibition in glioma using available chemical inhibitors of CSE. Propargylglycine (PAG) (Supplementary Figure S3a) is a nonproteinogenic amino acid that irreversibly blocks CSE through the double interaction with CSE and its cofactor pyridoxal 5-phosphate, which confers strong specificity to the PAG-CSE bond.^26,27^ We first performed a time course experiment in 2 IDH1m AS cell lines (NCH1681, NCH551b) with different concentrations of the inhibitor (10, 4, 2, 1, 0 mM) and of cysteine (360, 60, 30 µM) (Supplementary Figure S3b). At 360 µM cysteine, glioma cells were insensitive to PAG even at the highest concentration tested (10 mM), excluding significant off-target effects of the compound. Toxicity increased over time under cysteine-low conditions (60–30 µM) in line with the CSE KD experiments (Supplementary Figure S3b). Interestingly, the cytotoxic effect of PAG was specific to IDHm AS cells, and no significant toxicity was observed in PAG-treated IDHwt GBM cells (NCH644 and NCH421k) (Figure 4A). Again the effect was limited to low cysteine (≤60 µM) (Figure 4B).

To test if the dependency of IDH1m AS cells on CSE activity was related to their antioxidant capacity, we measured ROS levels in IDH1m and IDHwt cells. In line with the cytotoxicity, ROS increased in one of the PAG-treated IDH1m AS cells, although this was not robust in the second cell line (Supplementary Figure S3d). Taken together, these data provide evidence that CSE inhibition under limited cysteine selectively affects IDH1m AS, possibly mediated by increased oxidative stress.

### CSE Inhibition Delays Tumor Growth In Vivo

**Figure 5.**
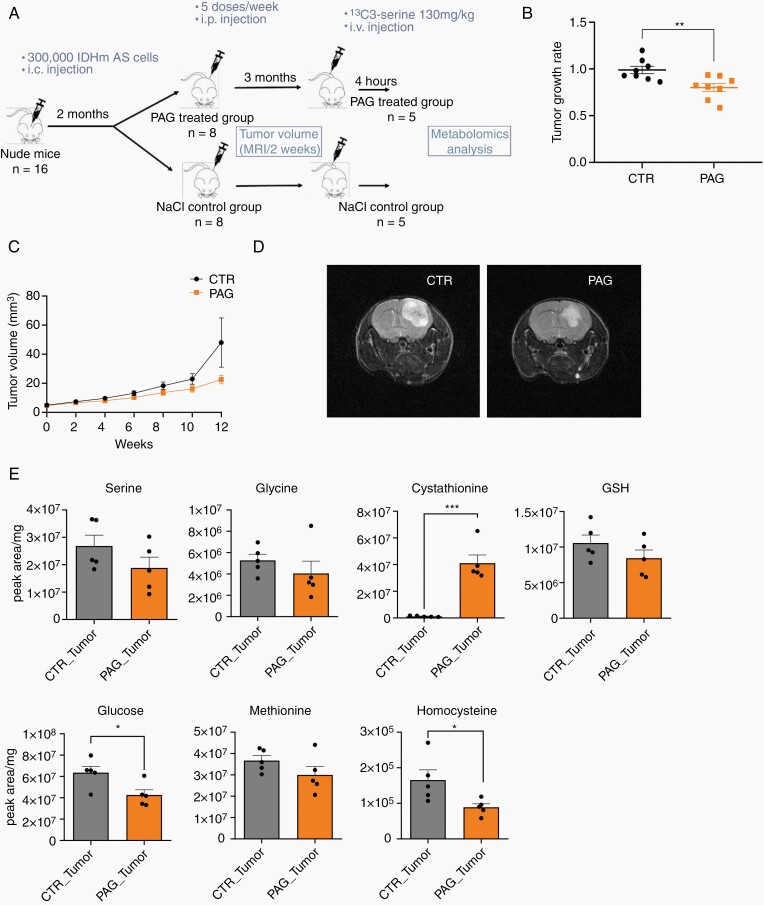
CSE inhibition delays tumor growth in vivo. (A) Setup of in vivo experiment. (B) Tumor growth rate of CTR and PAG-treated mice during the first 8 weeks of treatment (*n* = 8). (C) Tumor volume over time during the 3 months treatment period as determined by MRI (*n* = 8; except for CTR, where *n* = 7 at weeks 8/10 and *n* = 6 at week 12). Tendency to reduced volume at week 12 (not significant). Note that 2 CTR mice died after week 8. (D) Representative MRI pictures at week 12. (E) Total metabolite levels represented as normalized peak area in CTR and PAG-treated tumors after 12 weeks of treatment (*n* = 5). Each dot in charts B and E correspond to different mice. Data presented as means ± SEM. *P < .05; **P < .01; ***P < .001.

To evaluate the efficacy of CSE inhibition on tumor growth in vivo, we implanted IDH1m AS cells (NCH1681) into the brain of mice. After 2 months, tumors were detectable by MRI (around 5 mm^3^), mice were randomized into saline and PAG (35 mg/kg, 5 times/week) treated groups (Figure 5A). Treatment continued for 3 months and tumor size was monitored by bi-weekly MRIs. Within the first 2 months of treatment we found a significant decrease in tumor growth rate in PAG-treated mice (*n* = 8) (Figure 5B). A relative difference in tumor size was observed between both groups over time, which became pronounced, though not significant at the end of the third month (Figure 5C and D). The experiment was stopped when 2 mice of the control group reached clinical endpoint (Supplementary Figure S4a).

To determine the effect of PAG at the metabolite level, labeled ^13^C_3_-serine (130 mg/kg) was terminally infused in the tail vein (*n* = 2/group) (Figure 5A), reaching steady state at 4–6 h in the circulation and in the brain (Supplementary Figure S4b). Consistent with the CSE KD experiment, PAG-treated brain tumors showed a slight decrease in serine and glycine when compared with the control group (Figure 5E). Moreover, the total level of cystathionine was considerably increased in PAG-treated tumors (Figure 5E), demonstrating target engagement in vivo. This was accompanied by some decrease of methionine and a significant decrease of homocysteine (Figure 5E), both precursors of cystathionine. In line with the KD results, GSH and glucose levels were reduced in PAG-treated tumors (Figure 5E). A clear accumulation of cystathionine in PAG-treated mice was also observed in the liver, where de novo GSH biosynthesis normally occurs,^28^ while most other metabolic differences appeared to be tumor-specific (Supplementary Figure S4c). The isotopologue distribution revealed that unlabeled serine decreased in treated tumors, whereas ^13^C_3_-serine (M+3) showed a significant increase (Supplementary Figure S4d). Again, labeled serine only accounted for ~10% of the total pool, reflected in the small amount of labeled cystathionine (M+3) in contrast to the strong accumulation of unlabeled one (M+0) (Supplementary Figure S4d), suggesting preferential use of compensatory glucose-derived serine of IDH1m AS tumors also in vivo. In summary, we show that PAG inhibits CSE activity in IDH1m AS brain tumors which causes a significant delay in tumor growth, providing a rationale for a therapeutic potential of CSE inhibitors in IDH1m astrocytomas.

## Discussion

**Figure 6.**
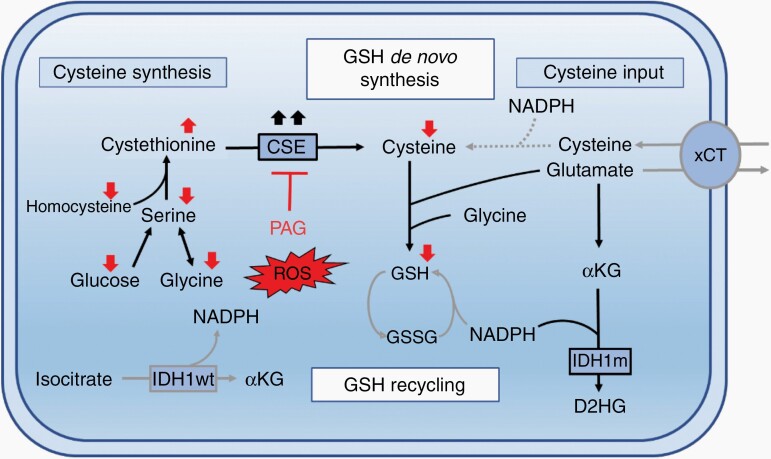
IDH1-mutant astrocytoma critically depend on CSE for GSH synthesis to counteract ROS. Schematic summary showing reliance of IDH1m astrocytomas on de novo GSH synthesis through increased cysteine production via CSE, thereby compensating for reduced GSH recycling due to limited NADPH (see text for details).

Due to the central role of IDH1 in NADPH recycling,^4,29^ several groups including ours suggested that IDH1m tumors may display vulnerabilities in redox metabolism and GSH production.^5,6,10,30,31^ Nevertheless, to date, there is limited data in part due to the difficulties of establishing IDHm cellular and animal models relevant to human disease. Our data, based on patient specimen and patient-derived cell lines with the endogenous IDH mutation indicates that CSE, the only known enzyme capable of synthesizing cysteine, is specifically upregulated in IDH1m AS and is essential to maintain GSH production under limited cysteine availability in vitro and in vivo (Figure 6). Inhibition of CSE reduced tumor growth in vivo, thus uncovering a novel druggable metabolic vulnerability in this aggressive glioma subtype.

Our proteomics analysis identified CSE as the most upregulated protein in IDH1m tumors. The importance of CSE as a source of cysteine for GSH production is well established in astrocytes^20,21^ and an early study in C6 glioma cells showed that CSE expression increases upon GSH depletion.^32^ However in IDHwt GBM, the expression of CSE is variable, suggesting a more plastic response to cysteine depletion, which is consistent with the specific sensitivity to CSE inhibition that we found in IDHm versus IDHwt cells. The limited expression of CSE in ODs can be explained by the location of its gene *CTH* on chromosomal arm 1p. In line with this, cystathionine, the substrate of CSE, was found to accumulate in IDH1m ODs,^33^ suggesting an alternative path to GSH production in these tumors.

We show that in addition to IDH status, the availability of extracellular cysteine determines the susceptibility to CSE inhibition, in line with recent data where CBS was shown to support cell growth during cysteine depletion.^34^ In the brain, where the uptake of amino acids is regulated by the blood brain barrier, the cysteine concentration is reportedly very low.^21,25,35–37^ Cysteine may be obtained via CSE-dependent synthesis or by uptake through the xCT antiporter exchanging glutamate for cystine. Several studies suggest a role for xCT to counteract ROS in IDHwt GBM^38–40^ and xCT inhibitors such as sulfasalazine have been explored in clinical trials against GBM with little outcome.^41,42^ Yet sulfasalazine is highly toxic and affects multiple other pathways.^43,44^ The importance of xCT in IDHm gliomas remains to be determined. Because of the low glutamate levels observed in IDHm gliomas (Figure 1B and ref. ^10^) and the requirement of reducing equivalents for the cystine-to-cysteine conversion,^36^ xCT activity may well be hampered in these tumors,^36^ potentially increasing the reliance on CSE. On the other hand, the fact that PAG did not significantly reduce GSH in tumors in the brain, despite efficient target engagement, suggests that alternative GSH pathways are active.

Despite the modest effect of PAG in vivo, it should be noted that a minor inhibition of growth in a slow growing tumor might well result in a valuable clinical outcome. Moreover, our data provide a rationale for combinatorial approaches, for example, the addition of PAG may increase the vulnerability to oxidative damage caused by radiation.^7^ Recently, the combination of radiotherapy with a glutaminase inhibitor was shown to increase mouse survival.^45^ The anti-tumor effects of CSE inhibition may be potentiated by a dietary restriction of cysteine to simultaneously interfere with exogenous and endogenous cysteine supply. This approach was well tolerated and more effective in depleting GSH in the brain of healthy rats compared to PAG alone.^46^ In other cancer models, the enzyme cyst(e)inase was found to efficiently degrade circulating cysteine, thereby increasing oxidative damage and decreasing tumor load.^47^ In conclusion, we here identified a specific dependency of IDH1m astrocytomas on CSE to maintain antioxidant homeostasis suggesting pharmacological inhibition of CSE as a potential strategy that warrants further investigation.

## Supplementary Material

vdab057_suppl_Supplementary_Materials

## Notes

**Authorship Statement.** Conceptualization: S.P.N., F.F., and A.C.G.; Methodology: A.C.G., A.O., S.P.N., D.S., S.T., J.M., E.M.G., and G.D.; Investigation: A.C.G., A.O., F.F., M.F.A., E.M.G., A.C.H., and A.D.F.; Formal analysis: A.C.G., J.M., M.F.A., D.S., and E.M.G.; Writing – Original Draft: A.C.G. and S.P.N.; Writing – Review and Editing: S.P.N., A.C.G., S.T., and J.M.; Funding acquisition: S.P.N., S.T., and J.M.; Resources: S.P.N., C.H.M., R.B., G.D., J.M., and S.T.; Supervision: S.P.N., J.M., and S.T.

## References

[1] Parsons DW , Jones S, Zhang X, et al. An integrated genomic analysis of human glioblastoma multiforme. Science. 2008;321(5897):1807–1812. 18772396 10.1126/science.1164382 PMC2820389

[2] Louis DN , Perry A, Reifenberger G, et al. The 2016 World Health Organization classification of tumors of the central nervous system: a summary. Acta Neuropathol. 2016;131(6):803–820. 27157931 10.1007/s00401-016-1545-1

[3] Dang L , White DW, Gross S, et al. Cancer-associated IDH1 mutations produce 2-hydroxyglutarate. Nature. 2009;462(7274):739–744. 19935646 10.1038/nature08617 PMC2818760

[4] Jiang L , Shestov AA, Swain P, et al. Reductive carboxylation supports redox homeostasis during anchorage-independent growth. Nature. 2016;532(7598):255–258. 27049945 10.1038/nature17393 PMC4860952

[5] Bleeker FE , Atai NA, Lamba S, et al. The prognostic IDH1(R132) mutation is associated with reduced NADP+-dependent IDH activity in glioblastoma. Acta Neuropathol. 2010;119(4):487–494. 20127344 10.1007/s00401-010-0645-6 PMC2841753

[6] Shi J , Sun B, Shi W, et al. Decreasing GSH and increasing ROS in chemosensitivity gliomas with IDH1 mutation. Tumour Biol. 2015;36(2):655–662. 25283382 10.1007/s13277-014-2644-z

[7] Juratli TA , Lautenschläger T, Geiger KD, et al. Radio-chemotherapy improves survival in IDH-mutant, 1p/19q non-codeleted secondary high-grade astrocytoma patients. J Neurooncol. 2015;124(2):197–205. 26033545 10.1007/s11060-015-1822-1

[8] Li K , Ouyang L, He M, et al. IDH1 R132H mutation regulates glioma chemosensitivity through Nrf2 pathway. Oncotarget. 2017;8(17):28865–28879. 28427200 10.18632/oncotarget.15868 PMC5438698

[9] Reitman ZJ , Jin G, Karoly ED, et al. Profiling the effects of isocitrate dehydrogenase 1 and 2 mutations on the cellular metabolome. Proc Natl Acad Sci USA. 2011;108(8):3270–3275. 21289278 10.1073/pnas.1019393108 PMC3044380

[10] Fack F , Tardito S, Hochart G, et al. Altered metabolic landscape in IDH-mutant gliomas affects phospholipid, energy, and oxidative stress pathways. EMBO Mol Med. 2017;9(12):1681–1695. 29054837 10.15252/emmm.201707729 PMC5709746

[11] Trong PD , Jungwirth G, Yu T, et al. Large-Scale drug screening in patient-derived idh(mut) glioma stem cells identifies several efficient drugs among FDA-approved antineoplastic agents. Cells. 2020;9(6):1389. 10.3390/cells9061389 PMC7348988 32503220

[12] Dao Trong P , Rösch S, Mairbäurl H, et al. Identification of a prognostic hypoxia-associated gene set in IDH-mutant glioma. Int J Mol Sci. 2018;19(10):2903. 10.3390/ijms19102903 PMC6212863 30257451

[13] Dettling S , Stamova S, Warta R, et al. Identification of CRKII, CFL1, CNTN1, NME2, and TKT as novel and frequent T-Cell targets in human IDH-Mutant Glioma. Clin Cancer Res. 2018;24(12):2951–2962. 29563135 10.1158/1078-0432.CCR-17-1839

[14] Bougnaud S , Golebiewska A, Oudin A, et al. Molecular crosstalk between tumour and brain parenchyma instructs histopathological features in glioblastoma. Oncotarget. 2016;7(22):31955–31971. 27049916 10.18632/oncotarget.7454 PMC5077988

[15] Bowman RL , Wang Q, Carro A, Verhaak RG, Squatrito M. GlioVis data portal for visualization and analysis of brain tumor expression datasets. Neuro Oncol. 2017;19(1):139–141. 28031383 10.1093/neuonc/now247 PMC5193031

[16] Tardito S , Oudin A, Ahmed SU, et al. Glutamine synthetase activity fuels nucleotide biosynthesis and supports growth of glutamine-restricted glioblastoma. Nat Cell Biol. 2015;17(12):1556–1568. 26595383 10.1038/ncb3272 PMC4663685

[17] Vande Voorde J , Ackermann T, Pfetzer N, et al. Improving the metabolic fidelity of cancer models with a physiological cell culture medium. Sci Adv. 2019;5(1):eaau7314. 30613774 10.1126/sciadv.aau7314 PMC6314821

[18] Meiser J , Tumanov S, Maddocks O, et al. Serine one-carbon catabolism with formate overflow. Sci adv. 2016;2(10):e1601273. 27819051 10.1126/sciadv.1601273 PMC5091358

[19] Golebiewska A , Hau AC, Oudin A, et al. Patient-derived organoids and orthotopic xenografts of primary and recurrent gliomas represent relevant patient avatars for precision oncology. Acta Neuropathol. 2020;140(6):919–949. 33009951 10.1007/s00401-020-02226-7 PMC7666297

[20] McBean GJ . The transsulfuration pathway: a source of cysteine for glutathione in astrocytes. Amino Acids. 2012;42(1):199–205. 21369939 10.1007/s00726-011-0864-8

[21] McBean GJ . Cysteine, glutathione, and thiol redox balance in astrocytes. Antioxidants (Basel). 2017;6(3):62. 10.3390/antiox6030062 PMC5618090 28771170

[22] Luchman HA , Chesnelong C, Cairncross JG, Weiss S. Spontaneous loss of heterozygosity leading to homozygous R132H in a patient-derived IDH1 mutant cell line. Neuro Oncol. 2013;15(8):979–980. 23757293 10.1093/neuonc/not064 PMC3714156

[23] Berg JM , Tymoczko JL, Stryer L.Biochemistry. 5th ed. New York: W H Freeman; 2002. Available from: https://www.ncbi.nlm.nih.gov/books/NBK21154/

[24] Psychogios N , Hau DD, Peng J, et al. The human serum metabolome. PLoS One. 2011;6(2):e16957. 21359215 10.1371/journal.pone.0016957 PMC3040193

[25] Engelborghs S , Marescau B, De Deyn PP. Amino acids and biogenic amines in cerebrospinal fluid of patients with Parkinson’s disease. Neurochem Res. 2003;28(8):1145–1150. 12834252 10.1023/a:1024255208563

[26] Sun Q , Collins R, Huang S, et al. Structural basis for the inhibition mechanism of human cystathionine gamma-lyase, an enzyme responsible for the production of H(2)S. J Biol Chem. 2009;284(5):3076–3085. 19019829 10.1074/jbc.M805459200

[27] Szabo C , Papapetropoulos A. International union of basic and clinical pharmacology. CII: pharmacological modulation of H2S levels: H2S donors and H2S biosynthesis inhibitors. Pharmacol Rev. 2017;69(4):497–564. 28978633 10.1124/pr.117.014050 PMC5629631

[28] Mosharov E , Cranford MR, Banerjee R. The quantitatively important relationship between homocysteine metabolism and glutathione synthesis by the transsulfuration pathway and its regulation by redox changes. Biochemistry. 2000;39(42):13005–13011. 11041866 10.1021/bi001088w

[29] Fan J , Ye J, Kamphorst JJ, Shlomi T, Thompson CB, Rabinowitz JD. Quantitative flux analysis reveals folate-dependent NADPH production. Nature. 2014;510(7504):298–302. 24805240 10.1038/nature13236 PMC4104482

[30] Tang X , Fu X, Liu Y, Yu D, Cai SJ, Yang C. Blockade of glutathione metabolism in IDH1-Mutated Glioma. Mol Cancer Ther. 2020;19(1):221–230. 31548295 10.1158/1535-7163.MCT-19-0103 PMC6946871

[31] Han S , Liu Y, Cai SJ, et al. IDH mutation in glioma: molecular mechanisms and potential therapeutic targets. Br J Cancer. 2020;122(11):1580–1589. 32291392 10.1038/s41416-020-0814-x PMC7250901

[32] Kandil S , Brennan L, McBean GJ. Glutathione depletion causes a JNK and p38MAPK-mediated increase in expression of cystathionine-gamma-lyase and upregulation of the transsulfuration pathway in C6 glioma cells. Neurochem Int. 2010;56(4):611–619. 20060865 10.1016/j.neuint.2010.01.004

[33] Branzoli F , Pontoizeau C, Tchara L, et al. Cystathionine as a marker for 1p/19q codeleted gliomas by in vivo magnetic resonance spectroscopy. Neuro Oncol. 2019;21(6):765–774. 30726924 10.1093/neuonc/noz031 PMC6556848

[34] Zhu J , Berisa M, Schwörer S, Qin W, Cross JR, Thompson CB. Transsulfuration activity can support cell growth upon extracellular cysteine limitation. Cell Metab. 2019;30(5):865–876.e5. 31607565 10.1016/j.cmet.2019.09.009 PMC6961654

[35] Bélanger M , Allaman I, Magistretti PJ. Brain energy metabolism: focus on astrocyte-neuron metabolic cooperation. Cell Metab. 2011;14(6):724–738. 22152301 10.1016/j.cmet.2011.08.016

[36] Paul BD , Sbodio JI, Snyder SH. cysteine metabolism in neuronal redox homeostasis. Trends Pharmacol Sci. 2018;39(5):513–524. 29530337 10.1016/j.tips.2018.02.007 PMC5912966

[37] Wang XF , Cynader MS. Astrocytes provide cysteine to neurons by releasing glutathione. J Neurochem. 2000;74(4):1434–1442. 10737599 10.1046/j.1471-4159.2000.0741434.x

[38] Singer E , Judkins J, Salomonis N, et al. Reactive oxygen species-mediated therapeutic response and resistance in glioblastoma. Cell Death Dis. 2015;6:e1601. 25590811 10.1038/cddis.2014.566 PMC4669764

[39] Chen L , Li X, Liu L, Yu B, Xue Y, Liu Y. Erastin sensitizes glioblastoma cells to temozolomide by restraining xCT and cystathionine-γ-lyase function. Oncol Rep. 2015;33(3):1465–1474. 25585997 10.3892/or.2015.3712

[40] Polewski MD , Reveron-Thornton RF, Cherryholmes GA, Marinov GK, Cassady K, Aboody KS. Increased expression of system xc- in glioblastoma confers an altered metabolic state and temozolomide resistance. Mol Cancer Res. 2016;14(12):1229–1242. 27658422 10.1158/1541-7786.MCR-16-0028 PMC6237285

[41] Takeuchi S , Wada K, Nagatani K, Otani N, Osada H, Nawashiro H. Sulfasalazine and temozolomide with radiation therapy for newly diagnosed glioblastoma. Neurol India. 2014;62(1):42–47. 24608453 10.4103/0028-3886.128280

[42] Robe PA , Martin D, Albert A, Deprez M, Chariot A, Bours V. A phase 1-2, prospective, double blind, randomized study of the safety and efficacy of Sulfasalazine for the treatment of progressing malignant gliomas: study protocol of [ISRCTN45828668]. BMC Cancer. 2006;6:29. 16448552 10.1186/1471-2407-6-29 PMC1368982

[43] Weber CK , Liptay S, Wirth T, Adler G, Schmid RM. Suppression of NF-kappaB activity by sulfasalazine is mediated by direct inhibition of IkappaB kinases alpha and beta. Gastroenterology. 2000;119(5):1209–1218. 11054378 10.1053/gast.2000.19458

[44] Wahl C , Liptay S, Adler G, Schmid RM. Sulfasalazine: a potent and specific inhibitor of nuclear factor kappa B. J Clin Invest. 1998;101(5):1163–1174. 9486988 10.1172/JCI992 PMC508669

[45] McBrayer SK , Mayers JR, DiNatale GJ, et al. Transaminase inhibition by 2-hydroxyglutarate impairs glutamate biosynthesis and redox homeostasis in glioma. Cell. 2018;175(1):101–116.e25. 30220459 10.1016/j.cell.2018.08.038 PMC6219629

[46] Cho ES , Hovanec-Brown J, Tomanek RJ, Stegink LD. Propargylglycine infusion effects on tissue glutathione levels, plasma amino acid concentrations and tissue morphology in parenterally-fed growing rats. J Nutr. 1991;121(6):785–794. 2033464 10.1093/jn/121.6.785

[47] Cramer SL , Saha A, Liu J, et al. Systemic depletion of L-cyst(e)ine with cyst(e)inase increases reactive oxygen species and suppresses tumor growth. Nat Med. 2017;23(1):120–127. 27869804 10.1038/nm.4232 PMC5218918

