# Supplementary material for: Cystathionine-γ-lyase drives antioxidant defense in cysteine-restricted IDH1-mutant astrocytomas
Source: Neurooncol Adv. 2021 Apr 9;3(1):vdab057. doi: 10.1093/noajnl/vdab057 (PMC8262642; doi:10.1093/noajnl/vdab057)
Supplement: vdab057_suppl_Supplementary_Materials [file vdab057_suppl_supplementary_materials.pdf]

SUPPLEMENTARY DATA

Cystathionine-γ-lyase drives antioxidant defense in cysteine-restricted IDH1 mutant astrocytomas

Neuro-Oncology Advances

Andrés Cano-Galiano[1], Anais Oudin[1], Fred Fack[1], Maria-Francesca Allega[3,4], David Sumpton[3,] Elena Martinez-Garcia[2], Gunnar Dittmar[2], Ann-Christin Hau[1]; Alfonso De Falco[5], Christel Herold-Mende[6], Rolf Bjerkvig[7,1]; Johannes Meiser[8*], Saverio Tardito[3,4*] and Simone P. Niclou[1,7]

[1]NORLUX Neuro-Oncology Laboratory, Department of Oncology, Luxembourg Institute of Health, Luxembourg;

[2]Quantitative biology unit, Luxembourg Institute of Health, Luxembourg;

[3]Cancer Research UK Beatson Institute, Glasgow, UK;

[4]Institute of Cancer Sciences, University of Glasgow, Glasgow, UK;

[5]National Center of Genetics, Laboratoire national de santé, Dudelange, Luxembourg;

[6]Department of Neurosurgery, University of Heidelberg, Germany;

[7]Department of Biomedicine, University of Bergen, Norway;

[8]Cancer Metabolism Group, Department of Oncology, Luxembourg Institute of Health, Luxembourg

* equal contribution

Corresponding author:

Simone P. Niclou: simone.niclou@lih.lu

SUPPLEMENTARY FIGURES

SUPPLEMENTARY FIGURE LEGENDS

SUPPLEMENTARY TABLES

SUPPLEMENTARY TABLE LEGENDS

SUPPLEMENTARY METHODS

SUPPLEMENTARY REFERENCES

Figure S1

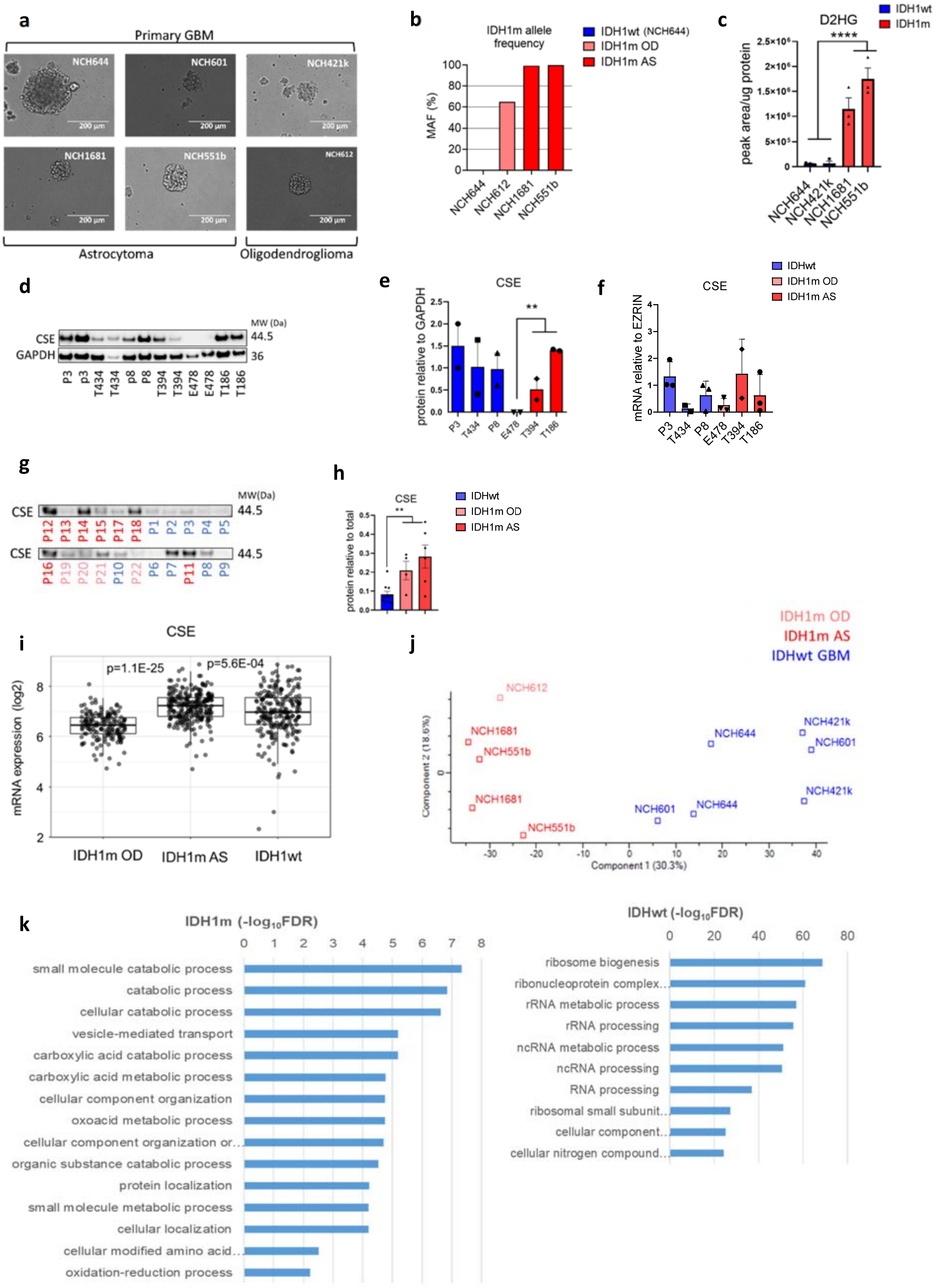

a
Primary GBM
NCH644
NCH601
NCH421k
NCH1681
NCH551b
NCH612
200 µm
Astrocytoma
Oligodendroglioma
b
IDH1m allele frequency
IDH1wt (NCH644)
IDH1m OD
IDH1m AS
MAF (%)
NCH644
NCH612
NCH1681
NCH551b
c
D2HG
IDH1wt
IDH1m
****
peak area/ug protein
NCH644
NCH421k
NCH1681
NCH551b
d
MW (Da)
CSE
GAPDH
44.5
36
P3 p3 T434 T434 p8 P8 T394 T394 E478 E478 T186 T186
e
CSE
**
protein relative to GAPDH
P3 T434 P8 E478 T394 T186
f
CSE
IDHwt
IDH1m OD
IDH1m AS
mRNA relative to EZRIN
P3 T434 P8 E478 T394 T186
g
MW(Da)
CSE
44.5
P12 P13 P14 P15 P17 P18 P1 P2 P3 P4 P5
P16 P19 P20 P21 P10 P22 P6 P7 P11 P8 P9
h
CSE
**
IDHwt
IDH1m OD
IDH1m AS
protein relative to total
i
CSE
p=1.1E-25
p=5.6E-04
mRNA expression (log2)
IDH1m OD
IDH1m AS
IDH1wt
j
IDH1m OD
IDH1m AS
IDHwt GBM
NCH612
NCH1681
NCH551b
NCH644
NCH421k
NCH601
Component 2 (18.6%)
Component 1 (30.3%)
k
IDH1m (-log10FDR)
small molecule catabolic process
catabolic process
cellular catabolic process
vesicle-mediated transport
carboxylic acid catabolic process
carboxylic acid metabolic process
cellular component organization
oxoacid metabolic process
cellular component organization or...
organic substance catabolic process
protein localization
small molecule metabolic process
cellular localization
cellular modified amino acid...
oxidation-reduction process
IDHwt (-log10FDR)
ribosome biogenesis
ribonucleoprotein complex...
rRNA metabolic process
rRNA processing
ncRNA metabolic process
ncRNA processing
RNA processing
ribosomal small subunit...
cellular component...
cellular nitrogen compound...

Figure S2

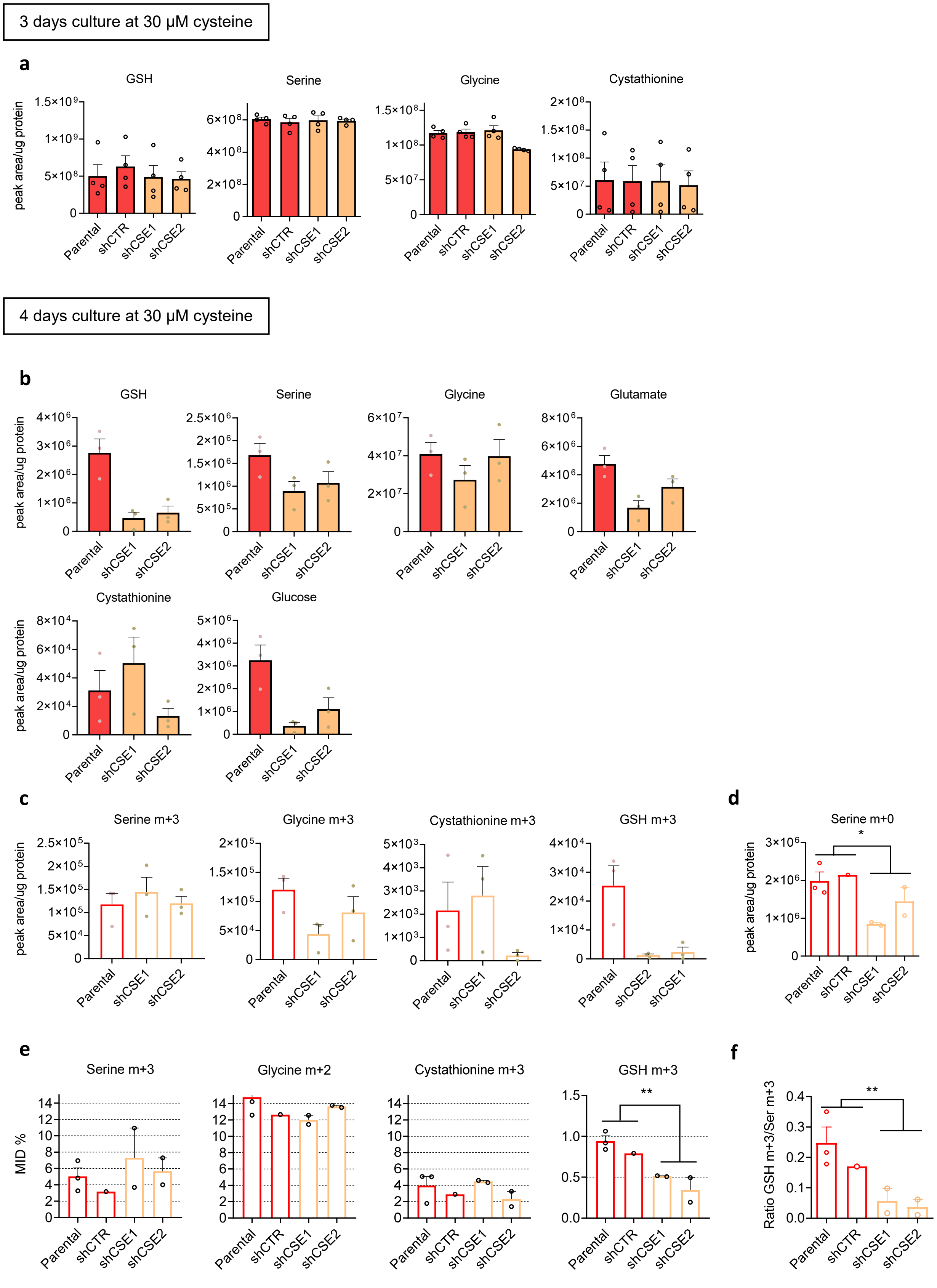

3 days culture at 30 μM cysteine
a
GSH
Serine
Glycine
Cystathionine
peak area/ug protein
Parental
shCTR
shCSE1
shCSE2
4 days culture at 30 μM cysteine
b
GSH
Serine
Glycine
Glutamate
Cystathionine
Glucose
peak area/ug protein
Parental
shCSE1
shCSE2
c
Serine m+3
Glycine m+3
Cystathionine m+3
GSH m+3
peak area/ug protein
d
Serine m+0
peak area/ug protein
*
e
Serine m+3
Glycine m+2
Cystathionine m+3
GSH m+3
MID %
**
f
Ratio GSH m+3/Ser m+3
**

Figure S3

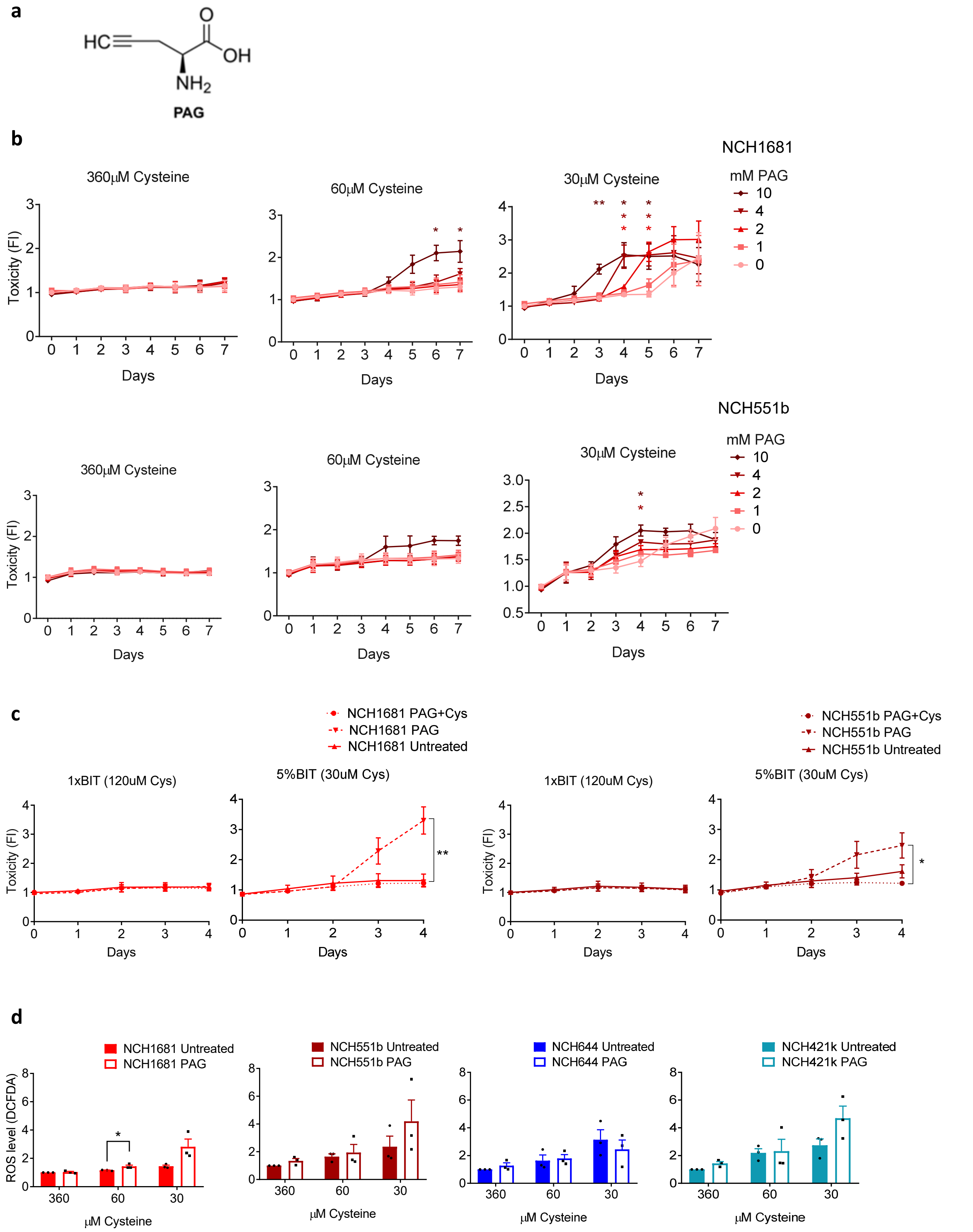
a
HC
O
OH
NH2
PAG
b
NCH1681
mM PAG
10
4
2
1
0
360µM Cysteine
60µM Cysteine
30µM Cysteine
Toxicity (FI)
Days
NCH551b
mM PAG
10
4
2
1
0
360µM Cysteine
60µM Cysteine
30µM Cysteine
Toxicity (FI)
Days
c
NCH1681 PAG+Cys
NCH1681 PAG
NCH1681 Untreated
NCH551b PAG+Cys
NCH551b PAG
NCH551b Untreated
1xBIT (120uM Cys)
5%BIT (30uM Cys)
Toxicity (FI)
Days
d
NCH1681 Untreated
NCH1681 PAG
NCH551b Untreated
NCH551b PAG
NCH644 Untreated
NCH644 PAG
NCH421k Untreated
NCH421k PAG
ROS level (DCFDA)
µM Cysteine

Figure S4

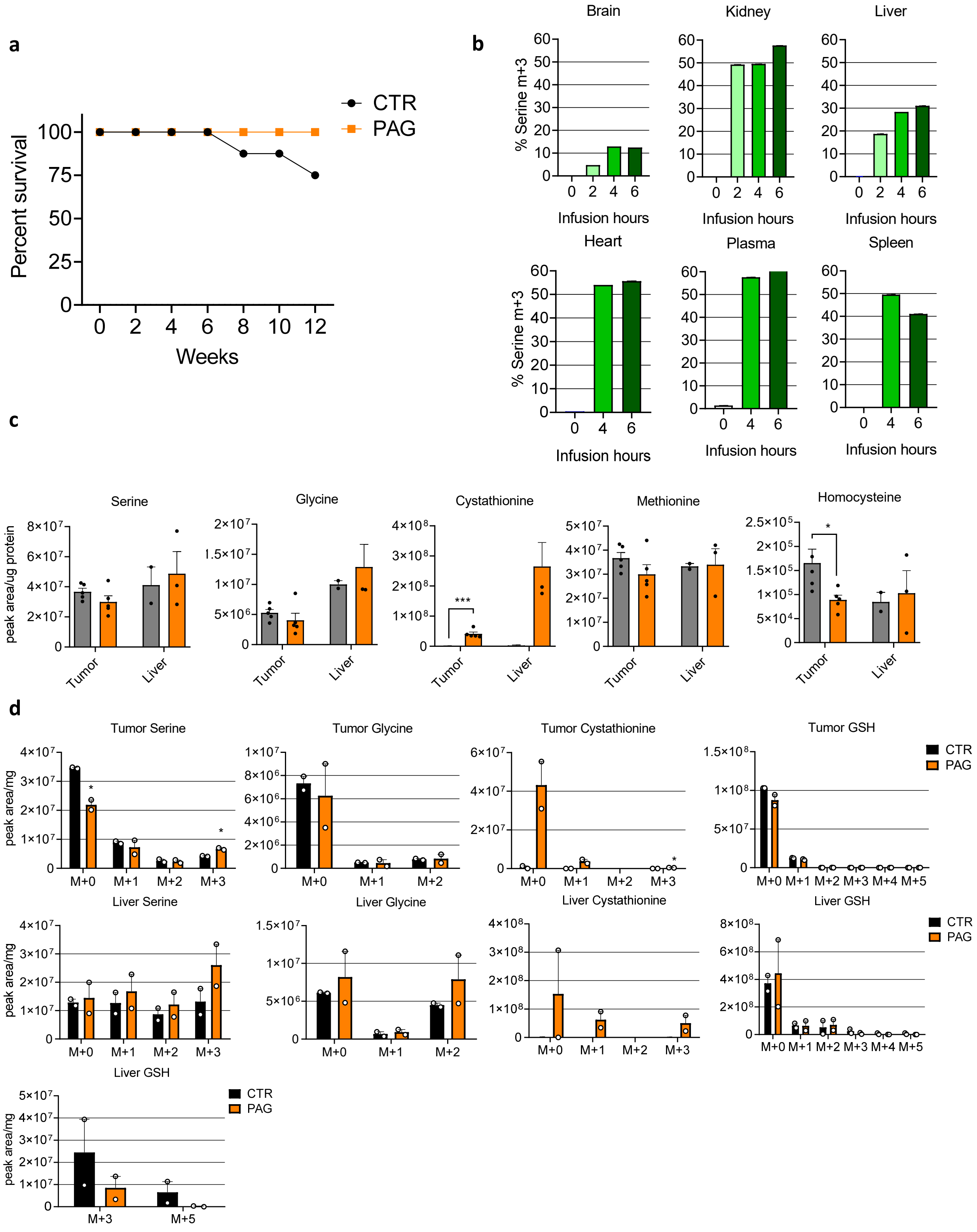

a
b
c
d
CTR
PAG
Percent survival
Weeks
Brain
Kidney
Liver
Heart
Plasma
Spleen
% Serine m+3
Infusion hours
Serine
Glycine
Cystathionine
Methionine
Homocysteine
peak area/ug protein
Tumor
Liver
Tumor Serine
Tumor Glycine
Tumor Cystathionine
Tumor GSH
Liver Serine
Liver Glycine
Liver Cystathionine
Liver GSH
peak area/mg
M+0
M+1
M+2
M+3
M+4
M+5

LEGENDS TO SUPPLEMENTARY FIGURES

Figure S1. Relative to Figure 1

(a) Phase contrast microscope imaging of the six patient derived glioma cell lines. NCH644, NCH601 and NCH421k correspond to patients with IDH1wt primary glioblastoma (GBM); NCH1681 and NCH551b correspond to patients with IDH1m astrocytoma; NCH612 correspond to a patient with IDH1m oligodendroglioma.

(b) Percentage of IDH1 mutant allele frequency (MAF) observed by digital PCR in all IDH1m cell lines, including astrocytomas (AS) (NCH551b, NCH1681) and oligodendroglioma (OD) (NCH612) and one of the IDHwt cell lines (NCH644). MAF and 95% confidence interval (CI) for each cell line were: NCH644 MAF= 0.00032%, CI= 8.59E-3% -- 0.117%; NCH612 MAF= 64.85%, CI= 61.196% -- 68.65%; NCH1681 MAF= 99.45%, CI= 93.223% -- 106%; NCH551b MAF= 99.98%, CI= 94.808% -- 105.33%. Digital PCR procedure was performed as described in[1]. Error bars correspond to 95% confidence intervals (CI) of MAF estimation. One cell stock replicate for each line (n=1).

(c) D2HG and GSH levels in patient-derived IDH1wt glioblastoma and IDH1m astrocytoma cell lines as determined by LC-MS. Each biological replicate (n) correspond to an independent cell culture stock of each cell line (n=3).

(d) Western blot of CSE in patient-derived orthotopic xenografts: IDH1wt GBM (P3, T434, P8), IDH1m OD (E478) and IDHm AS (T394, T186). MW (Da) represents the specific molecular weights of each protein. (e) Quantification thereof relative to GAPDH. Each biological replicate (n) correspond to an indepenedent xenograft tumor (n=2). Original blots are shown in Figure 1SC.

(f) *CSE* gene expression in patient-derived orthotopic xenografts relative to *EZRIN* determined by qPCR. Each biological replicate (n) correspond to an independent xenograft tumor (n=3, except for T434 and T394 where n=2).

(g) Western blot of clinical samples for CSE. IDHwt (blue; P1-P10), IDH1m AS (red; P11-P18) and IDH1m OD (pink; P19-P22). (h) Quantification thereof relative to total protein amount. Each biological replicate (n) correspond to an independent patient tumor, IDHwt (n=10), IDH1m AS (n=6) and IDH1m OD (n=4).

(i) *CSE* gene expression in glioma patients (source: Gliovis [2]). IDH1m OD (169; 25.76%), IDH1m AS (258; 39.33%), IDH1wt (229; 34.91%). TCGA_GBMLGG adult glioma dataset. Pairwise t tests. Pairwise comparisons between group levels with corrections for multiple testing (p-values with Bonferroni correction).

(j) Principal Component Analysis (PCA) of the untargeted proteomics comparison between IDH1m AS (red) IDH1m OD (pink) and IDHwt GBM (blue) cell lines

GBM: Glioblastoma; OD: Oligodendroglioma; AS: Astrocytoma

(k) Gene Ontology Biological Process (GOBP) terms representation of the top 10 terms (IDHwt) and top 15 (IDH1m) terms, based on STRING database analysis [3].

Data in A and F presented as means ± SEM. ** $p<0.01$; **** $p<0.0001$.

Figure S2. **Relative to Figure 3**

(a) Total GSH, serine, glycine and cystathionine levels in parental, shCTR, shCSE1 and shCSE2. Cells were incubated during 3 days at 30 µM cysteine. Each dot of the bar charts correspond to an independent experiment (n=4).

(b) Total GSH, serine, glycine, glutamate cystathionine and glucose levels in parental, shCSE1 and shCSE2. Cells were incubated during 4 days at 30 µM cysteine.

(c) Isotopologue levels of GSH, serine, glycine and cystathionine after $^{13}C_3$-serine tracing (ratio 1:1 natural L-Serine and L-Serine13C3) in parental, shCSE1 and shCSE2. One independent experiment represented in (b) and (c) (n=1). Each dot corresponds to a technical replicate. Three

technical replicates in each group. No statistical analysis was applied due to the lack of biological replicates

(d) Natural serine (m+0) levels of parental, shCTR, shCSE1 and shCSE2 cells incubated during 4 days with 30 µM cysteine and $^{13}C_3$-serine tracing. Each dot of the bar charts correspond to an independent experiment (n). Parental (n=3), shCTR (n=1), shCSE1 (n=2) and shCSE2 (n=2).

(e) Mass Isotopologue Distribution (MID) percentages of serine m+3, glycine m+2, cystathionine m+3 and GSH m+3in CSE knockdown and control cells. Cells were incubated during 4 days at 30 µM cysteine. Each dot of the bar charts correspond to an independent experiment (n). Parental (n=3), shCTR (n=1), shCSE1 (n=2) and shCSE2 (n=2). (f) GSH m+3/Ser m+3 ratio of same cells as in (d).

Data presented as means ± SEM.* $p<0.05$ ** $p<0.01$

Figure S3. Relative to Figure 4

(a) Chemical structure of PAG.

(b) Toxicity of IDH1m astrocytoma cell lines (NCH1681 and NCH551b) incubated with different cysteine concentrations (360, 60 and 30 µM respectively) and with different PAG concentrations (0, 1, 2, 4 and 10 mM) over time. Data of toxicity presented as fold change relative to 360 µM cysteine medium, day 0 untreated (n=3).

(c) Validation of *in vitro* cysteine titration via BSA Insulin Transferring (BIT) supplement dilution (Detailed explanation in methods section “Cysteine titration mediums”). Toxicity of IDH1m astrocytoma cell lines (NCH1681 and NCH551b) incubated with 20% and 5%BIT expecting to obtain 120 µM and 30 µM cysteine respectively. The toxicity of CSE inhibitor PAG at 5%BIT is rescued by addition of extra cysteine up to 120 µM, which matches with the lack of toxicity at 20%BIT and the expected 120 µM cysteine. Data presented as fold change relative to 20% BIT (120µM) at day 0 untreated (n=3).

(d) ROS production upon PAG (10mM) and at different cysteine concentrations, presented as fold change of DCFDA fluorescence intensity (FI) relative to control cells (untreated, 360µM cysteine) (n=3).

N represents one independent experiment. Data presented as means ± SEM. * $p<0.05$; ** $p<0.01$

Figure S4. Relative to Figure 5

(a) Mass Isotopologue Distribution (MID) percentage of heavy labelled $^{13}C_3$-serine (serine m+3) incorporated in different tissues after tracer infusion over time (n=1).

(b) Total serine, glycine, cystathionine, GSH, glucose, methionine and homocysteine levels in tumor and liver with and without PAG treatment (tumor CTR (n=5), tumor PAG (n=5), liver CTR (n=2), liver PAG (n=3)). Data presented as means ± SEM.

(c) Total isotopologue levels of serine, glycine, cystathionine and GSH in tumor and in liver with and without PAG treatement (n=2). Data presented as means ± SEM. * $p<0.05$; ** $p<0.01$

SUPPLEMENTARY TABLES

Supplementary Table 1

Glioma group	Patient	Age	Gender	Histopathological diagnosis	Molecular diagnosis-Methylation Class	IDH status
IDH wt	P1	38	Male	GBM	NA	IDHwt*
	P2	65	Male	GBM	GBM, mesenchymal	IDHwt
	P3	56	Male	GBM	NA	IDHwt*
	P4	71	Female	GBM	GBM, RTK II	IDHwt
	P5	51	Male	GBM	GBM, RTK I	IDHwt
	P6	69	Male	GBM	GBM, mesenchymal	IDHwt
	P7	73	Male	GBM	NA	IDHwt*
	P8	72	Male	GBM	GBM, mesenchymal	IDHwt
	P9	68	Female	GBM	NA	IDHwt*
	P10	26	Female	Oligodendroglioma	NA	IDHwt*
IDHm AS	P11	31	Male	GBM	High grade astrocytoma	IDH1m
	P12	37	Male	Astrocytic glioma	High grade astrocytoma	IDH1m
	P13	41	Male	Astrocytic glioma	High grade astrocytoma	IDH1m
	P14	23	Male	Astrocytic glioma	Astrocytoma	IDH1m
	P15	34	Male	Astrocytic glioma	Astrocytoma	IDH1m
	P16	37	Male	Oligodendroglioma	Astrocytoma	IDH1m
	P17	NA	NA	Astrocytic glioma	NA	NA
	P18	NA	NA	Astrocytic glioma	NA	NA
IDHm OD	P19	47	Male	Oligodendroglioma	Oligodendroglioma 1p/19q codeleted	IDH1m
	P20	47	Female	Oligodendroglioma	Oligodendroglioma 1p/19q codeleted	IDH1m
	P21	36	Male	Oligodendroglioma	Oligodendroglioma 1p/19q codeleted	IDH1m
	P22	51	Male	Oligodendroglioma	Oligodendroglioma 1p/19q codeleted	IDH1m

Supplementary Table 2

Culture medium	DMEMF12 (300 μM cys) *	DMEM cys-free	BIT100 (600 μM cys) *	Extra 30mM cys	Final [cys]
**Normal NCH cells**	80%**	-	20%	-	360 μM
**Cysteine titration mediums**	-	80%	20%	1%	360 μM
	-	80%	20%	-	120 μM
	-	90%	10%	-	60 μM
	-	95%	5%	-	30 μM

LEGENDS TO SUPPLEMENTARY TABLES

Supplementary Table 1. Clinical sample characteristics and glioma classification.

Methylation class according to Heidelberg classifier. NA: not available. * IDH status of samples which methylation diagnosis was not available, were determined by digital PCR

Supplementary Table 2. Cell culture media with different cysteine concentrations

* Cys concentrations refer to the combination of both cysteine (reduced) and cystine (oxidized). DMEMF12: 300 μM cys = 100 μM reduced and 100 μM oxidiced. BIT100: 600 μM cys = 300 μM oxidized. **Percentage in volume over the total volume of the mix

Supplementary Table 3 (see related excel table). Differentially expressed proteins in IDHm and IDHwt cell lines.

List with all the 127 proteins upregulated in IDHm cell lines (NCH1681, NCH551b, NCH612) and 258 proteins upregulated in IDHwt cell lines (NCH644, NCH421k, NCH601), according to **Figure 1I,J** (False discovery rate (FDR)<0.05; Fold change (FC)<2).

SUPPLEMENTARY METHODS

Clinical samples

Clinical samples for LC-MS analysis were obtained from 22 patients (10 IDHwt glioblastomas; 8 IDH1m astrocytomas; 4 IDH1m oligodendrogliomas) from the Haukeland Hospital, Bergen, Norway. Tissue fragments were stereotactically sampled (25mg) during the operation and snap-frozen. Diagnosis was first determined by classical histopathology, followed by molecular diagnosis through DNA methylation profiling (Infinium® MethylationEPIC, Illumina) as described [1]. In brief, genomic DNA was extracted using the QIAamp Blood & Tissue Kit. Bisulfite conversion was done using up to 250ng of extracted gDNA. DNA methylation-based glioma classification was performed by referencing data to the dataset of over 2800 neuropathological tumors at https://www.molecularneuropathology.org/mnp as described previously [4] . For samples where methylation profiling was not possible due to limited sample amount, IDH status was confirmed by digital PCR as previously described [1]. Patient characteristics are shown at **Supplementary Table 1**. Patient sample collection was approved by the local ethics committee (Haukeland University Hospital, Bergen (REK 2010/130-2) and in line with the Declaration of Helsinki. Statistical analysis in patient samples was performed using the mean and SEM of biological replicates (n), meaning all tumors from the same group (IDHwt GBM, IDH1m AS or IDH1m OD)

Patient-derived glioma cell lines and xenografts

Patient-derived cell lines were generated in the laboratory of Dr Christel Herold-Mende and grown as 3D spheres under serum-free conditions (**Figure S1a**). These glioma stem-like cells (GSCs) have been previously used in other studies [5-8]. NCH644, NCH601 and NCH421k correspond to patients with IDH1wt Glioblastoma. NCH1681 and NCH551b were from patients with IDH1m astrocytoma; NCH612 from a patient with an IDH1m Oligodendroglioma. All IDH1m cells were slow growing and sensitive to culture conditions. For routine culture, all cells were grown as non-adherent spheres in DMEM-F12 medium (Lonza, BE04-687F/U1) supplemented with 20% BIT100 (Provitro), 2mM L-Glutamine, 30 U/ ml Pen-Strep, 1U/ml Heparin (Sigma), 20 ng/ml bFGF (Miltenyi, 130-093-041), and 20ng/ml EGF (Provitro, 1325950500). Cells were incubated under standard normoxic conditions at 37°C and 5% CO_2 and were regularly tested for mycoplasma. The presence of the IDH mutation was regularly validated by Western blot and by digital PCR as previously described[1]. Glioma patient-derived orthotopic xenografts (PDOX) generated in NOD/Scid mice (male or female, at least 2 months of age) were described previously [1,9].

Western blot

Cells were lysed in RIPA extraction buffer supplemented with protease inhibitor cocktail (Roche). Protein quantification was performed with 2D-Quant (GE Healthcare). Protein lysates were resolved in NuPage 4-12 % BisTris gels (ThermoFisher), and blotted onto PVDF membranes. Membranes were blocked with TBST buffer in 2% milk. Blots were incubated with primary antibodies at 4°C overnight with: anti-IDH1m R132H (DIANOVA DIA-H09), anti-Cystathionase (Abcam ab189916), anti-GAPDH (Cell Signaling 5174S) and anti-LaminB1 (Abcam ab16048). Secondary antibodies anti-mouse and anti-rabbit (Jackson ImmunoResearch) were applied, and blots were developed with a chemiluminescent substrate (ThermoFisher) using ImageQuant analyzer (GE Healthcare). Control antibodies anti-GAPDH and anti-LAMINB1 were used in the same blot as experimental antibodies for normalized quantification. Statistical analysis was performed using the mean and SEM of all biological replicates (n). For cell lines, n represents

protein samples extracted from different cell culture stocks. For xenografts, n represents protein samples extracted from different mouse tumors.

Quantitative RT-PCR

RNA was isolated following the Trizol protocol (ThermoFisher) and cDNA was synthesized using iScript Reverse Transcriptase (BioRad). cDNA was applied for real-time PCR reactions in a Via7 instrument using Fast SYBR Green (Applied Biosystems). Relative gene expression levels were normalized against elongation factor 1 alpha (*EF1α*) housekeeping gene, and calculated using the ΔCT method. All reactions were performed in triplicate wells and the mean was used per data point. Specific primers used were: *CTH* (Fw: GGCCTGGTGTCTGTTAATTGT, Rv: GCCATTCCGTTTTTGAAATGCT), *CBS* (Fw: GGCCAAGTGTGAGTTCTTCAA, Rv: GGCTCGATAATCGTGTCCCC), *EF1α* (Fw: TTGTCGTCATTGGACACGTAG, Rv: TGCCACCGCATTTATAGATCAG), *EZRIN* (Fw: TGCCCCACGTCTGAGAATC, Rv: CGGCGCATATACAACTCATGG) (Eurogentec). Statistical analysis was performed using the mean and SEM of all biological replicates (n). For cell lines, n represents cDNA samples extracted from different cell culture stocks. For xenografts, n represents cDNA samples extracted from different xenograft tumors. All biological replicates from all different groups were analyzed at the same time in one PCR run.

TCGA analysis

Gene expression data from glioma patients was obtained from The Cancer Genome Atlas (TCGA) dataset via the GlioVis data portal [2].

Untargeted proteomics analysis by LC-MS/MS

For the proteomic analysis, all 6 cell lines were extracted by two different preparation methods for a total of two biological replicates (n) per cell line (except for NCH612 where only one replicate

could be analysed). Each biological replicate (n) represents protein samples extracted from different cell culture stocks. In the first preparation, proteins were extracted using methanol/chloroform and protein pellets were dissolved in 8M urea buffer, while in the 2nd preparation the cells were lysed with 1% sodium deoxycholate detergent (SDC). Protein concentrations were determined by EZQ assay (Invitrogen, cat#R33200). Equal amounts of proteins were used for the analysis. Samples were reduced with 5 mM dithiothreitol for 1 h at 37°C and alkylated with 15 mM iodoacetamide for 30 min at RT followed by a two-step sequential protein digestion with Lys-C (Wako, cat#125-05061) for 3 h at 37°C, and trypsin (Promega, cat#V5111) at 37°C overnight. Tryptic peptides were desalted on C18 SPE (Sep Pak tC18, 25 mg, Waters) and dried using a vacuum centrifuge. Peptides were analyzed on a Q-Exactive HF mass spectrometer (Thermo Scientific) coupled with a Dionex Ultimate 3000 RSLC chromatography system operated in column switching mode. Peptides were trapped on a 75 µm x 2 cm pre-column (C_{18} pepmap 100, 3 µm) and separated onto a 75 µm × 50 cm column (C_{18} pepmap 100, 2 µm) by a 240 min linear gradient starting from 2 % solvent A (0.1% formic acid in water) to 35% solvent B (0.1% formic acid in 100 % acetonitrile) at a flow rate of 300 nl/minute. The mass spectrometer was operated in a data-dependent acquisition mode with a survey scan acquired at a resolving power of 120,000 at 200m/z. The 12 most intense multiple-charged ions ($z \geq 2$) were isolated and fragmented at normalized collision energy of 28 and the resulting fragment ions acquired at a resolution of 15,000 at 200 m/z. Fragmented precursors m/z were excluded for another fragmentation for 20 s. MS files were analyzed in the MaxQuant [10] software version 1.6.7.0. MS/MS spectra were searched by the Andromeda search engine [11] against the TrEMBL UniProt Homo sapiens (February 2019, 73,928 entries). Cysteine carbamidomethylation was set as a fixed modification and methionine oxidation and N-terminal protein acetylation as variable modifications. For protein quantification, label-free quantitation (LFQ) was performed with a minimum ratio count of 2 [12] with match between runs activated. The FDR cut-off for peptide and protein identifications was set to 1%. Bioinformatic analysis was performed in the Perseus

platform version 1.6.2.1 (Tyanova et al, 2016). Two-sample Student's t-test was used to determine the significantly changed proteins between IDH1wildtype and IDH1mutant cell lines with a permutation-based FDR of 0.05 for multiple testing correction. Results were filtered to have both a significant FDR-corrected P-value and a minimum fold change of 2.

Generation of stable shRNA-mediated knockdown cell lines

Since patient-derived glioma cells were not amenable to standard transfection procedures, all gene knockdown (KD) experiments were performed with lentiviral vectors to establish stably transduced KD lines. To target the *CSE* gene in NCH1681 cells, two different shRNA variants were used: shCSE1 (ATAGCTTTAGGTTCTTGAG; Code: V2LHS_151012) and shCSE2 (ATGAAAGATAATGAGGTGC; Code: V2LHS_151015), and one non-targeting scrambled shRNA was used as control: shCTR (CTTACTCTCGCCCAAGCGAGAG; Code: RHS4346) (Dharmacon). Plasmids containing shRNAs and GFP (**Figure S2C**) (pGIPZ-shRNA-targets and pGIPZ-shRNA-scramble) were amplified by midi-prep (Machery-Nagel). Lentiviral particle production was done in HEK293T ($5x10^5$ cells in 5ml of DMEM 2% FBS) using the core packaging construct pCMVR8.74 and envelope protein vector pMD2.G. The supernatant containing viral particles was harvested and 1ml was used to transfect NCH1681 ($8x10^5$ cells in 3ml of DMF12 specific medium for IDH1m cells). After lentiviral transduction, cells were passaged five times in the presence of 1ug/ml puromycin as selection marker to obtain stable GFP-positive KD cells.

Cysteine titration in medium

The optimal culture medium for IDH1m cell lines is based on DMEMF12 (Lonza, BE04-687F/U1) (containing 100 µM cysteine and 100 µM of its oxidized dimer cystine) supplemented with 20% BIT100 (containing 600 µM cysteine), thus the final cysteine concentration (combining both cysteine and cystine) in the medium being 360 µM (**Table 1**). With cysteine-free DMEMF12 (see below) plus 20% BIT the final cysteine concentration was still at 120 µM. Since BIT contains

essential hormones for cell growth (transferrin, insulin and BSA), completely eliminating BIT was too deleterious for the sensitive IDHm cells. Therefore to further decrease the concentration of cysteine we applied serial BIT dilutions, at 10% BIT (60 µM cysteine) and 5% BIT (30 µM cysteine) (**Supplementary table 2**). We confirmed that the toxic effect of PAG in low BIT/cysteine medium was due to the lack of cysteine by rescuing the cells with extracellular cysteine (**Figure S4c**). The cysteine-free medium consisted of DMEM high glucose, no glutamine, no methionine, no cysteine (ThermoFisher, 21013024), supplemented with an amino acid mix to reach these final concentrations: 115 µM methionine, 250 µM glycine, 50 µM alanine, 50 µM asparagine, 50 µM aspartic acid, 50 µM glutamic acid, 50 µM proline, RPMI 1640 Vitamins Solution 1X (Sigma, R7256), 2mM L-Glutamine, 30 U/ml Pen-Strep, 1 U/ml Heparin (Sigma), 20 ng/ml bEGF (Miltenyi, 130-093-041), 20 ng/ml EGF (Provitro, 1325950500).

Sphere size measurement

Sphere growth assays were performed in an IncuCyte imaging incubator. Cells ($1x10^3$ cells/well) were seeded in a 384 well plate in 75 µl. The plate was briefly centrifuged (3 min, 300 rpm) and placed in the incubator at 37ºC to allow sphere formation. Twelve hours later, once the spheres were properly formed, each single sphere per well was imaged every 4 hours for 7 days. For quantification of sphere size a specific mask was applied (IncuCyte ZOOM 2018A software) and raw data were taken from Phase Object Confluence (POC), which measures the percentage of the phase contrast image area that is occupied by objects (cells). Five spheres were measured per cell line in each independent experiment. The mean of the five spheres was used as data point for each independent experiment. Statistical analysis was performed using mean and SEM of independent experiments (n).

GFP fluorescence as a proxy for cell viability

GFP fluorescence was measured in an IncuCyte imaging incubator. Cells ($1x10^3$ cells/well) were seeded in a 384 well plate in 50 µl. The plate was briefly centrifuged (3 min, 300 rpm) and placed in the incubator at 37ºC to allow sphere formation. Twelve hours later, the spheres were properly formed and each single sphere per well was imaged every day for 6 days. Five spheres were measured per cell line in each independent experiment. The mean of the five spheres was used as data point for each independent experiment. Statistical analysis was performed using mean and SEM of independent experiments (n). Raw data were taken from mean green fluorescent (MGF) values from IncuCyte ZOOM 2018A software. Statistical analysis was performed using mean and SEM of independent experiments (n). [5]

Toxicity assay

Toxicity was determined with the CellTox Green Cytotoxicity assay® (Promega). The CellTox working solution consists of a compound that emits green fluorescence after reacting with free extracellular DNA. Cells ($1x10^3$ cells/well) were seeded in a 384 well plate in 75 µl of the desired medium, the plate was briefly centrifuged (3 min, 300 rpm) and 25 µl of CellTox working solution were added. The plate was then placed in the incubator under normoxia at 37ºC. Twelve hours later, green fluorescence intensity (FI) was measured in a CLARIOstar Plus microplate reader (BMG LABTECH) and again every day for up to 4 days. Linear variable bandpass filter (*LVF*) 470-15; Auto 488.8; 515-20 nm. Five spheres were measured per cell line in each independent experiment. The mean of the five spheres was used as data point for each independent experiment. Statistical analysis was performed using mean and SEM of independent experiments (n).

ROS measurement

$1.5x10^5$ cells/ml/well were seeded in a 12 well plate. Each well contained a specific cysteine concentration medium (360, 60, 30 µM cysteine). The plate was placed in the incubator under

normoxia at 37ºC for 3 days to allow ROS formation. After 4 days, 2′,7′-Dichlorofluorescin diacetate (DCFDA) probe was applied at 10 µM. After 30 minutes, cells were washed, harvested in PBS and seeded in 8 replicate wells in a 96-well plate ($3x10^4$ cells/well in 100 µl). Green fluorescence was determined in a CLARIOstar Plus microplate reader (BMG LABTECH). Linear variable bandpass filter (*LVF*) 470-15; Auto 488.8; 515-20 nm. The mean of the 8 wells was used as data point for each independent experiment. Statistical analysis was performed using mean and SEM of independent experiments (n).

In vivo experiment in orthotopic xenografts

$3x10^5$ NCH1681 cells were stereotactically injected into the frontal cortex of NSG mice (NOD.Cg-$Prkdc^{scid}$ $Il2rg^{tm1Wjl}$/SzJ) (n=20). Tumor growth was monitored by MRI (FSE-T2 sequence, 3T MRI system, MR Solutions) as described previously [1]. MRI data were analyzed by ImageJ and the tumor volume (mm^3) was determined by tumor delineation in each slice and multiplying by slice thickness (1mm). Two months after implantation, mice with tumors (size range 2-10 mm^3) were selected and randomized into 2 groups (8 mice per group). The experimental group received intraperitoneal injections of PAG (35mg/kg), 5 times/week during 3 months, while the control group received saline injections. The treatment dose was based on previous reports [13]. Tumor growth rate (GR) was calculated using the tumor volume (TV) measurement at 2 different time points as GR = 100 * log (TVf/TV0) / (tf-t0), where TVf and TV0 are the tumor volumes at the last and first time points respectively, and tf-t0 is the difference in days between the time points [14]. Mice were observed daily, about two months after treatment start, mice from the control group started to present neurological symptoms and body weight loss. The experiment was halted 3 months post-treatment after two control mice had died.

For *in vivo* flux analysis, 130mg/kg/h of L-serine (U-13C3, 99%) (CLM-1574-H-0.1 Eurisotop) was intravenously (tail vein) infused in 2 mice of each group during 4 hours, as well as in 2 healthy mice without tumors (healthy brain controls). Mice were maintained anesthetized with 2.5% of

isoflurane during the infusion. The 4 hours and 130mg/kg concentration of serine tracer infusion which was based on [15], was confirmed in our pilot experiment where metabolite steady state in the brain ws reached after 4 hrs (**Figure S5b**). After infusion brain, liver, kidney and heart were dissected and flash frozen in isopentane at -80ºC LC-MS analysis

All procedures were approved by the animal welfare structure of LIH and the national authorities responsible for animal experiments in Luxembourg under the reference LUPA 2020/15.

Metabolite and flux analysis in cell lines and tissues

Cell lines ($5x10^5$ cells/well) were seeded in triplicate in 12 well plates in a total volume of 2ml in 30 µM cysteine. For the tracing experiments 400 µM of L-serine (U-13C3, 99%) (CLM-1574-H-0.1 Eurisotop) was added to the culture medium which already contained 400 µM natural serine (ThermoFisher, 21013024) (ratio $^{13}C3$-Serine/Serine 1:1). After 4 days, cells were washed in PBS and transferred to 1.5ml Eppendorf tubes. Same experiment was performed with metabolite extraction after 3 days. Cell lysis was done in 300 µl of cold metabolite extraction solvent (30% Acetonitril 50% MeOH 20%H2O). Samples were put on a shaker (1000 rpm at 4ºC for 5 minutes) and centrifuged at 4ºC for 10 minutes. 150 µl of supernatant was collected and stored at -80ºC for downstream LC-MS. The cell pellets were kept for protein quantification using the Lowry method for sample normalization. For the LC-MS analysis, the mean of three triplicate wells was used as data point for independent experiment (n). Statistical analysis was performed using mean and SEM of independent experiments (n).

Frozen tissue of human or mouse samples were cut with a scalpel on dry ice and weighed to obtain pieces of 15-25mg. Mousee tumor samples were carefully collected by cryomicrotome sectioning of the frozen brains. 25 µl/mg tissue of metabolite extraction buffer (30% Acetonitril 50% MeOH 20%H_2O) was added and samples were homogenized via bead-mill (Qiagen Tissuelyzer) mechanical disruption with 5 mm beads (2 times x 20 seconds shake at 20 Hz frequency). Samples were shaken at 1400 rpm (12 minutes at 4ºC) and centrifuged at 16000 g

(15 minutes at 4ºC). Metabolite extracts in the supernatant were recovered and stored at -80ºC until LC-MS analysis was performed.

A pilot GC-MS-based approach to determine steady state levels of $^{13}C_3$-serine in different mice tissues (**Figure S5b**) was performed as described in [16]. LC-MS analysis was performed as previously described (Vande Voorde et. al 2019 Science Advances). Briefly, analysis was carried out on an Ultimate 3000 HPLC coupled to Q Exactive Orbitrap mass spectrometer with electrospray (ESI) ionization (Thermo Scientific, Waltham, MA, USA). Metabolite extracts (5µl) were separated on ZIC-pHILIC column (SeQuant, 150 x 2.1mm, 5µm, Merck KGaA, Darmstadt, Germany) held at 45°C. The gradient started at 20% A (20mM ammonium carbonate, pH 9.2) and 80% B (acetonitrile), decreasing to 20% B at a flow rate of 200µL/min over 15 minutes. Data was acquired using Thermo Xcalibur software over a mass range of 75-1000 m/z at a resolution of 35,000 (at 200m/z) with polarity switching. Data analysis was carried out in TraceFinder (v4.0, Thermo Scientific) software. Metabolites were identified using an in-house library of exact mass and known retention time generated using commercial standards on the same LC-MS system.

Statistical analysis

All statistical analyses were performed using GraphPad Prism 8 software. Statistical comparisons were based on two-tailed Student's t-test between the corresponding groups, except where otherwise stated. Number of independent cell experiments or biological replicates (n), errors and p-values are indicated in each corresponding figure legend.

SUPPLEMENTARY REFERENCES

1 Golebiewska, A. *et al.* Patient-derived organoids and orthotopic xenografts of primary and recurrent gliomas represent relevant patient avatars for precision oncology. *Acta Neuropathol*, doi:10.1007/s00401-020-02226-7 (2020).

2 Bowman, R. L., Wang, Q., Carro, A., Verhaak, R. G. & Squatrito, M. GlioVis data portal for visualization and analysis of brain tumor expression datasets. *Neuro Oncol* **19**, 139-141, doi:10.1093/neuonc/now247 (2017).

3 Szklarczyk, D. *et al.* STRING v11: protein-protein association networks with increased coverage, supporting functional discovery in genome-wide experimental datasets. *Nucleic Acids Res* **47**, D607-d613, doi:10.1093/nar/gky1131 (2019).

4 Capper, D. *et al.* DNA methylation-based classification of central nervous system tumours. *Nature* **555**, 469-474, doi:10.1038/nature26000 (2018).

5 Trong, P. D. *et al.* Large-Scale Drug Screening in Patient-Derived IDH(mut) Glioma Stem Cells Identifies Several Efficient Drugs among FDA-Approved Antineoplastic Agents. *Cells* **9**, doi:10.3390/cells9061389 (2020).

6 Dao Trong, P. *et al.* Identification of a Prognostic Hypoxia-Associated Gene Set in IDH-Mutant Glioma. *Int J Mol Sci* **19**, doi:10.3390/ijms19102903 (2018).

7 Dettling, S. *et al.* Identification of CRKII, CFL1, CNTN1, NME2, and TKT as Novel and Frequent T-Cell Targets in Human IDH-Mutant Glioma. *Clin Cancer Res* **24**, 2951-2962, doi:10.1158/1078-0432.Ccr-17-1839 (2018).

8 Bougnaud, S. *et al.* Molecular crosstalk between tumour and brain parenchyma instructs histopathological features in glioblastoma. *Oncotarget* **7**, 31955-31971, doi:10.18632/oncotarget.7454 (2016).

9 Bougnaud, S. *et al.* Molecular crosstalk between tumour and brain parenchyma instructs histopathological features in glioblastoma. *Oncotarget* **7**, 31955-31971, doi:10.18632/oncotarget.7454 (2016).

10 Cox, J. & Mann, M. MaxQuant enables high peptide identification rates, individualized p.p.b.-range mass accuracies and proteome-wide protein quantification. *Nat Biotechnol* **26**, 1367-1372, doi:10.1038/nbt.1511 (2008).

11 Cox, J. *et al.* Andromeda: a peptide search engine integrated into the MaxQuant environment. *J Proteome Res* **10**, 1794-1805, doi:10.1021/pr101065j (2011).

12 Cox, J. *et al.* Accurate proteome-wide label-free quantification by delayed normalization and maximal peptide ratio extraction, termed MaxLFQ. *Mol Cell Proteomics* **13**, 2513-2526, doi:10.1074/mcp.M113.031591 (2014).

13 Cho, E. S., Hovanec-Brown, J., Tomanek, R. J. & Stegink, L. D. Propargylglycine infusion effects on tissue glutathione levels, plasma amino acid concentrations and tissue morphology in parenterally-fed growing rats. *J Nutr* **121**, 785-794, doi:10.1093/jn/121.6.785 (1991).

14 Mehrara, E., Forssell-Aronsson, E., Ahlman, H. & Bernhardt, P. Specific growth rate versus doubling time for quantitative characterization of tumor growth rate. *Cancer Res* **67**, 3970-3975, doi:10.1158/0008-5472.Can-06-3822 (2007).

15 Ducker, G. S. *et al.* Reversal of Cytosolic One-Carbon Flux Compensates for Loss of the Mitochondrial Folate Pathway. *Cell Metab* **23**, 1140-1153, doi:10.1016/j.cmet.2016.04.016 (2016).

16 Meiser, J. *et al.* Pro-inflammatory Macrophages Sustain Pyruvate Oxidation through Pyruvate Dehydrogenase for the Synthesis of Itaconate and to Enable Cytokine Expression. *J Biol Chem* **291**, 3932-3946, doi:10.1074/jbc.M115.676817 (2016).
